# Supplementary figures and images for: Feature architecture aware phylogenetic profiling indicates a functional diversification of type IVa pili in the nosocomial pathogen Acinetobacter baumannii
Source: PLoS Genet. 2023 Jul 27;19(7):e1010646. doi: 10.1371/journal.pgen.1010646 (PMC10374093; doi:10.1371/journal.pgen.1010646)

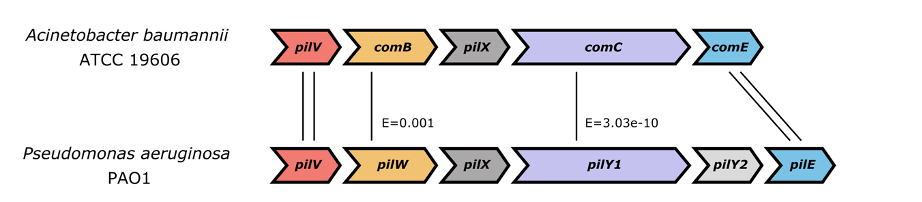

Supplement: S1 Fig — The individual genes are represented by arrows, where the arrow direction indicates the direction of transcription. Gene names are indicated within the boxes. The naming of A. baumannii genes follows [46] and the naming of P. aeruginosa genes follows [47]. Genes in the two species that are connected by two lines have been identified as orthologs (see Fig 1B in the main manuscript). Genes connected by a single line represent A. baumannii proteins that identify the P. aeruginosa protein as a best BlastP hit. The BlastP hit E-value is given next to the connecting line. Genes without a connection lack a significantly similar sequence in the respective other species. From conservation of gene order follows that comB–pilW, pilX–pilX, and comC–pilY1 are the corresponding orthologs in the two species. NCBI accession numbers: A. baumannii ATCC 19606T: pilV—WP_002194578.1; comB—WP_000079195.1; pilX—WP_086221418.1; comC—WP_085940514.1; comE—WP_001046417.1. P. aeruginosa PAO1: pilV—NP_253241.1; pilW—NP_253242.1; pilX—NP_253243.1; pilY1—NP_253244.1; pilY2—NP_253245.1; pilE—NP_253246.1. (PNG) [file pgen.1010646.s001.png]

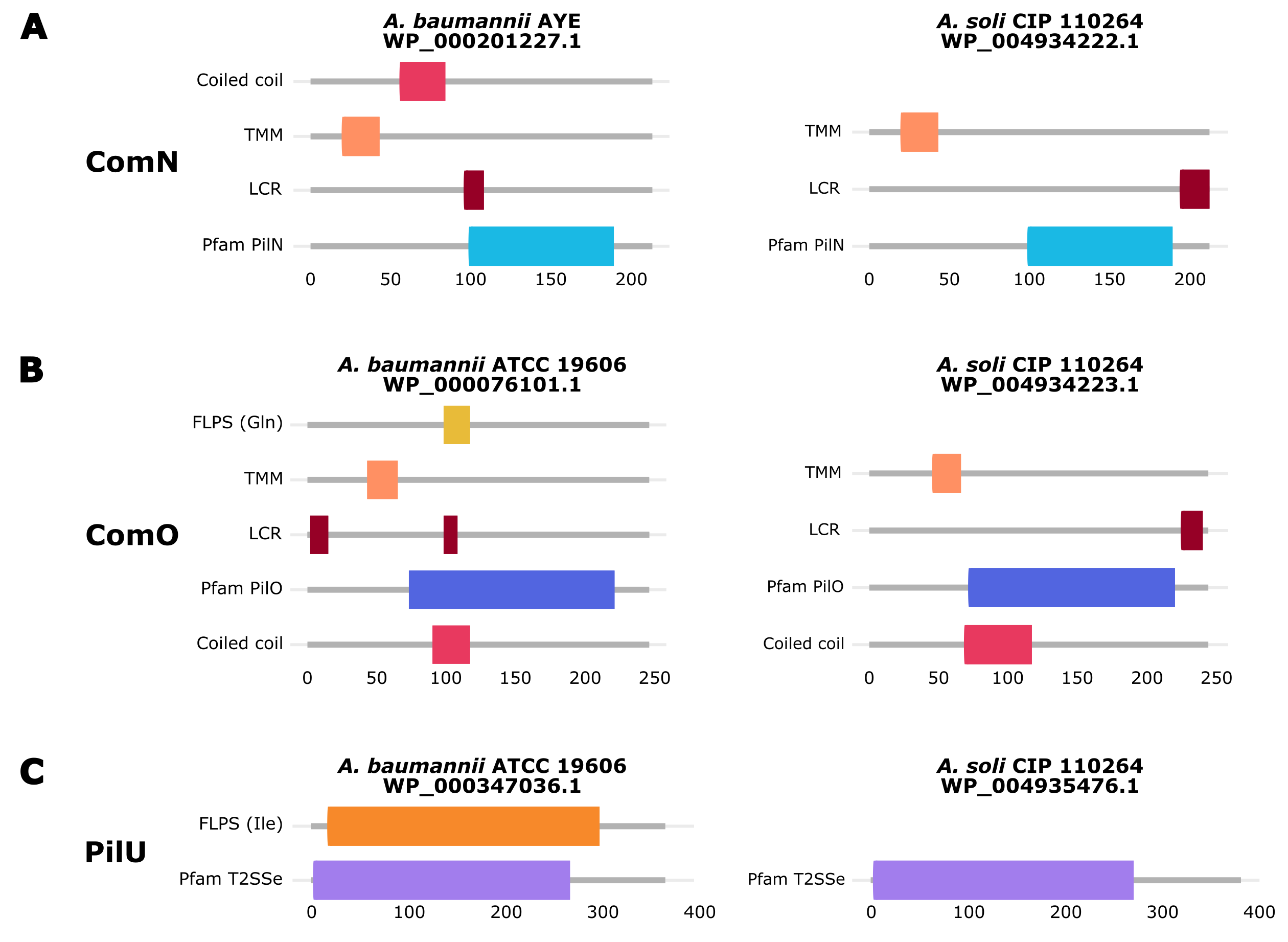

Supplement: S2 Fig — For the three represented components (A-C), the differences in domain architectures between the protein in Ab ATCC 19606T and their orthologs in other isolates are limited to coiled coil regions, low complexity regions (LCR) and regions with a compositional bias (FLPS) (see main text, Fig 1). Reference proteins shown in the first column are compared against their orthologs in Acinetobacter soli CIP 110264 (GCF_000368705.1). TMM–Transmembrane domain; Gln–Glutamine; Ile–Isoleucine. (PNG) [file pgen.1010646.s002.png]

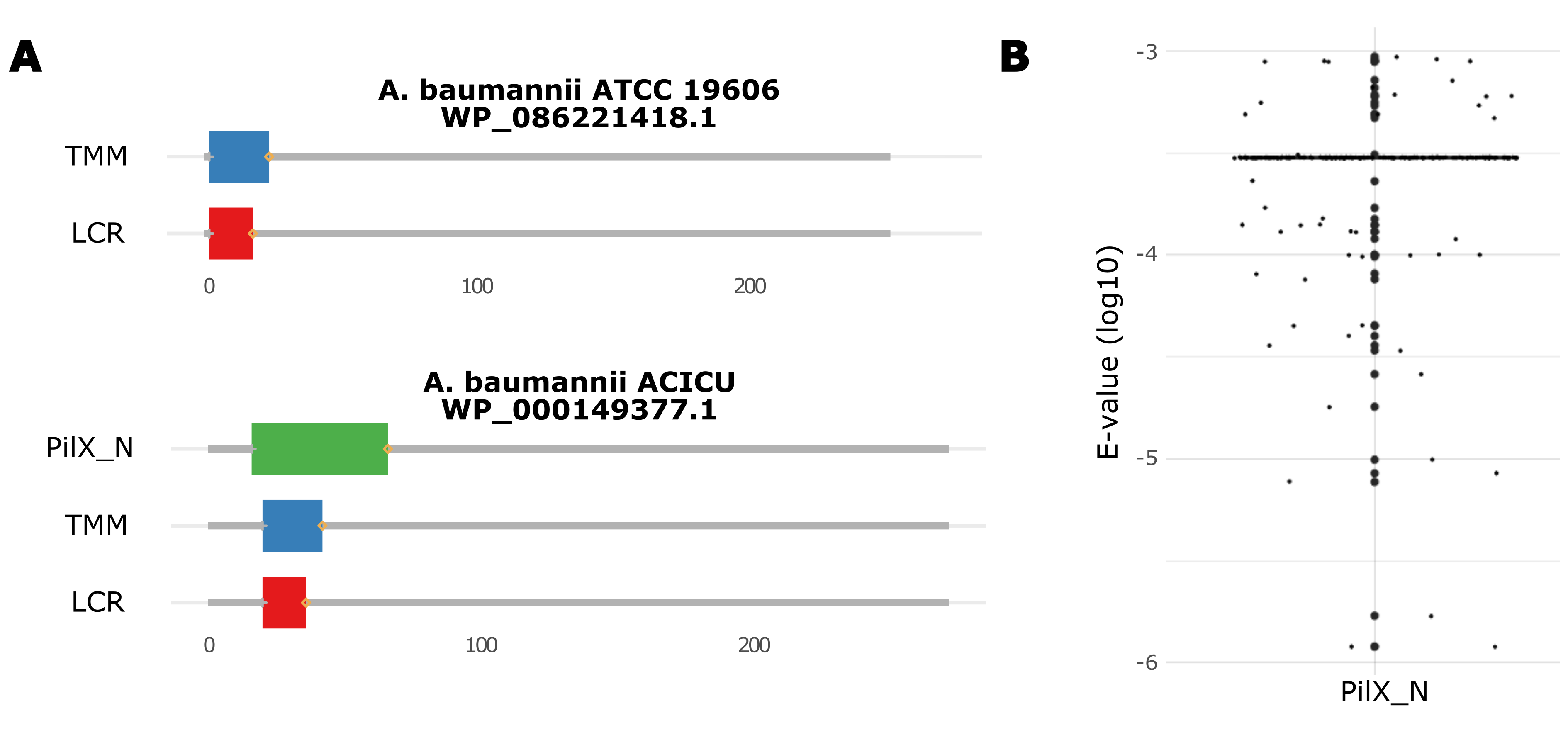

Supplement: S3 Fig — Only a subset of PilX orthologs was annotated with a PilX_N Pfam domain (PF14341) whereas the feature architectures of the remaining orthologs comprise only a transmembrane domain (TMM) and a low complexity region (LCR) (A). The box plot shows the E-value distribution for the annotated PilX_N Pfam domains. Most values are high and close to the inclusion threshold of 0.001. (PNG) [file pgen.1010646.s003.png]

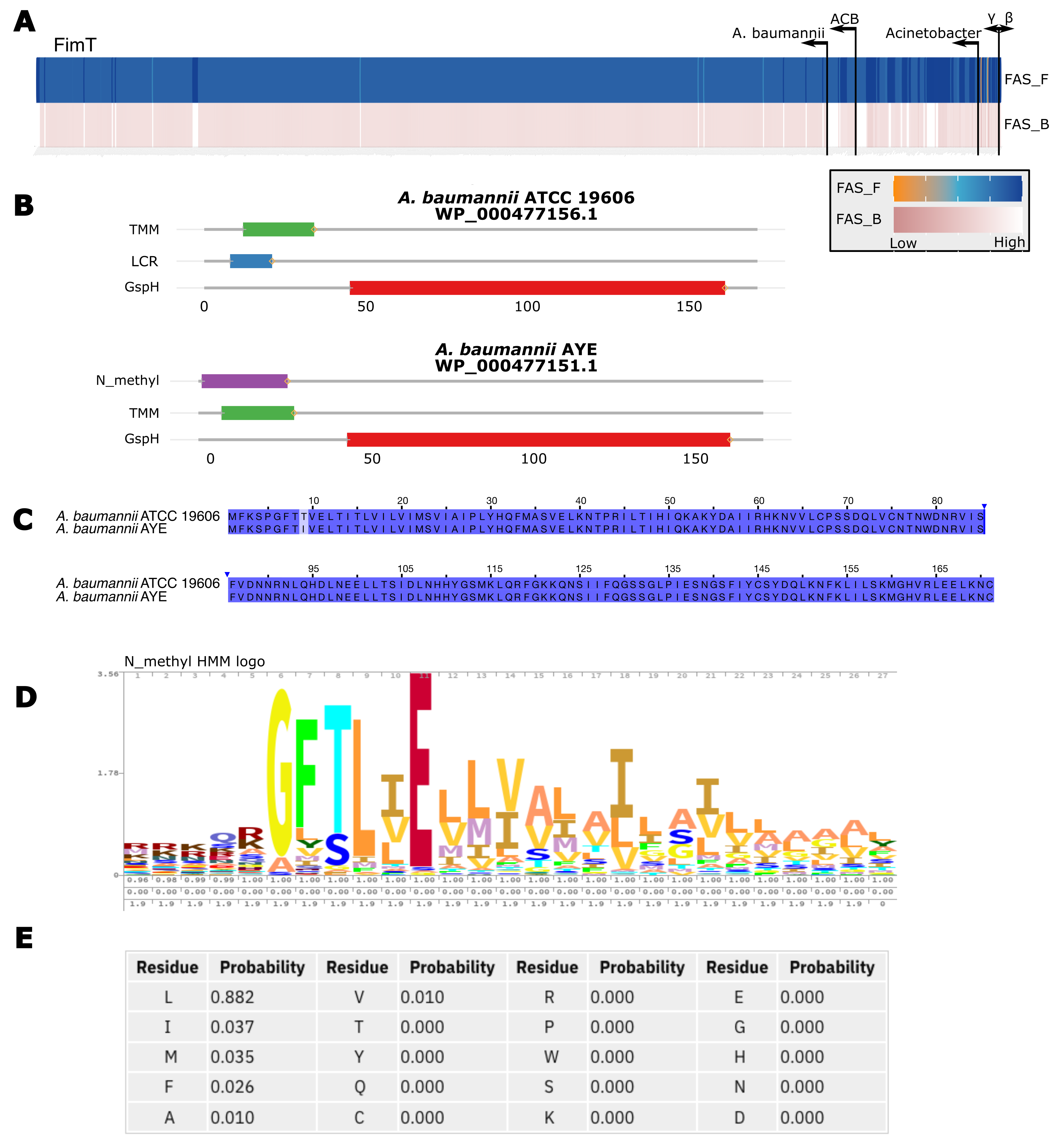

Supplement: S4 Fig — (A) Pair-wise feature architecture similarities between Ab ATCC 19606T FimT and its 864 orthologs across the analyzed taxa [68]. The upper block indicates the feature architecture similarity (FAS) scores penalizing the non-detection of an Ab ATCC 19606T feature in the ortholog (FAS_F). The consistent coloring in blue indicates that the overall domain architecture similarity is high. The lower block penalizes the absence of a feature that is seen in the ortholog but that is absent in Ab ATCC 19606T FimT (FAS_B). Most orthologs are colored in pink, which indicates that they possess a domain that is not annotated in Ab ATCC 19606 T FimT. (B) Pairwise feature architecture comparison between FimT in Ab ATCC 19606 T (WP_000477156.1) and its ortholog in Ab AYE (WP_000477147.1). This reveals that Ab ATCC 19606 T FimT could not be annotated with the N-terminal methylation motif provided by Pfam (PF07963; purple). (C) Pairwise sequence alignment between FimT in Ab ATCC 19606 T and in Ab AYE. Conserved residues are highlighted in dark blue; substitutions are shown in light blue. FimT of Ab ATCC 19606 T features a threonine at position 9 in the alignment. (D) The HMM weblogo of the N_methyl domain reveals that position 9 is typically occupied by either leucine or isoleucine. (E) Emission probabilities for the 20 amino acids at position 9 in the pHMM representing the Pfam N_methyl motif (PF07963). The emission probability for threonine is 0 at this position, which explains why no N_methyl domain was annotated in FimT of Ab ATCC 19606 T. ACB–Acinetobacter calcoaceticus-baumannii complex; γ–γ-proteobacteria; β–β-proteobacteria. (PNG) [file pgen.1010646.s004.png]

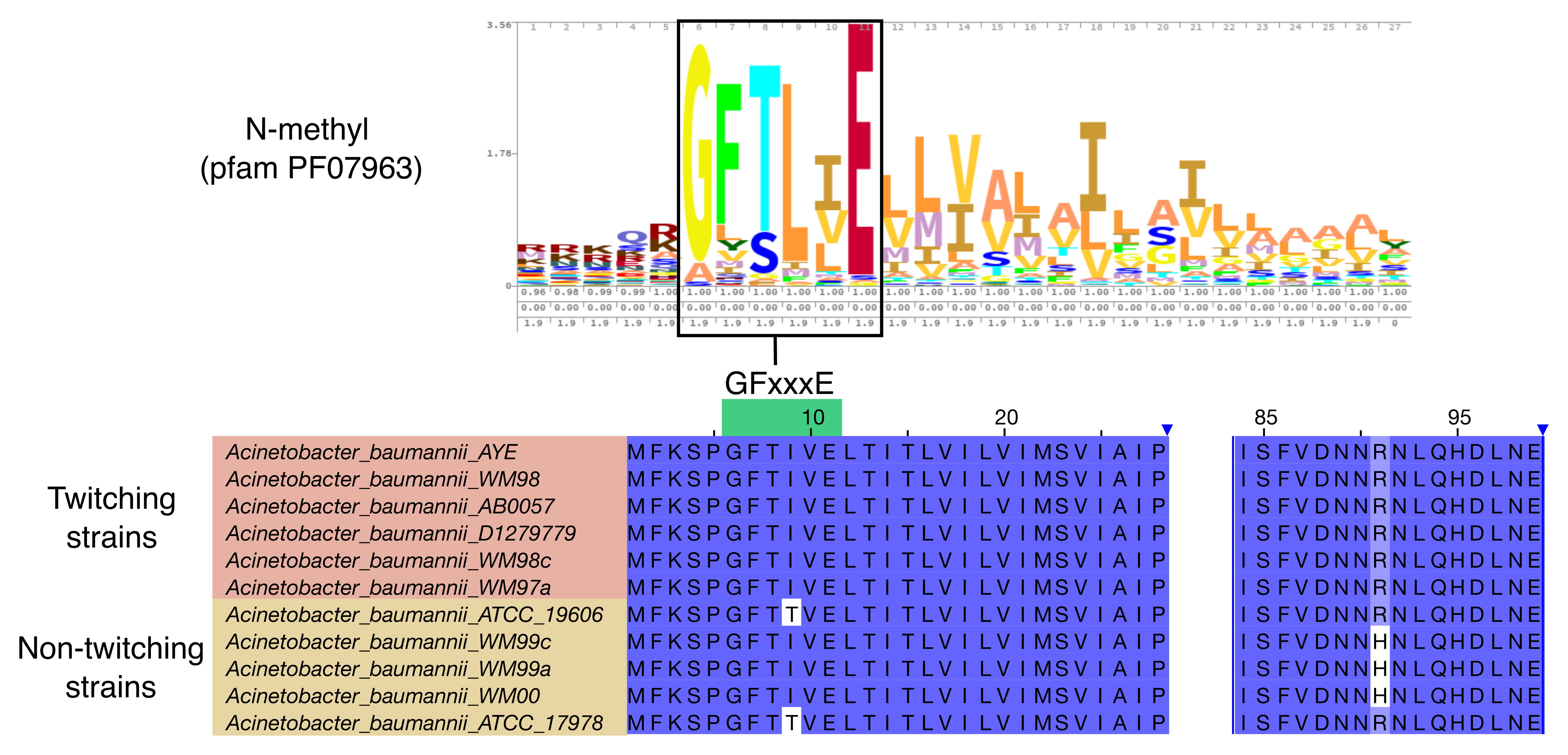

Supplement: S5 Fig — Twitching and non-twitching A. baumannii strains were taken from [10] and the corresponging FimT proteins were aligned. The multiple sequence alignment shows only the parts of the FimT alignment that differ between the investigated strains. The color code represents the degree of sequence conservation. The five non-twitching strains are characterized by two substitutions in FimT. Two strains, including Ab ATCC 19606T, display an I->T substitution at position 9 in the alignment. This position is part of a prepilin peptidase cleavage motif [49] indicated in green above the alignment. The consensus motif together with the corresponding part of the N-methyl Pfam pHMM is shown on top of the green box (see also S4 Fig). The I->T substitution likely alters the function of the cleavage motif such that it interferes with the processing of FimT. As a consequence, the ability to twitch is lost. Three further non-twitching strains show the canonical prepilin peptidase cleavage motif, however they share an R->H substitution at Pos. 91 in the alignment. Because this is the only difference of these strains to the twitching A. baumannii strains, it is tempting to speculate that also the R->H substitution is sufficient to abolish twitching. (PNG) [file pgen.1010646.s005.png]

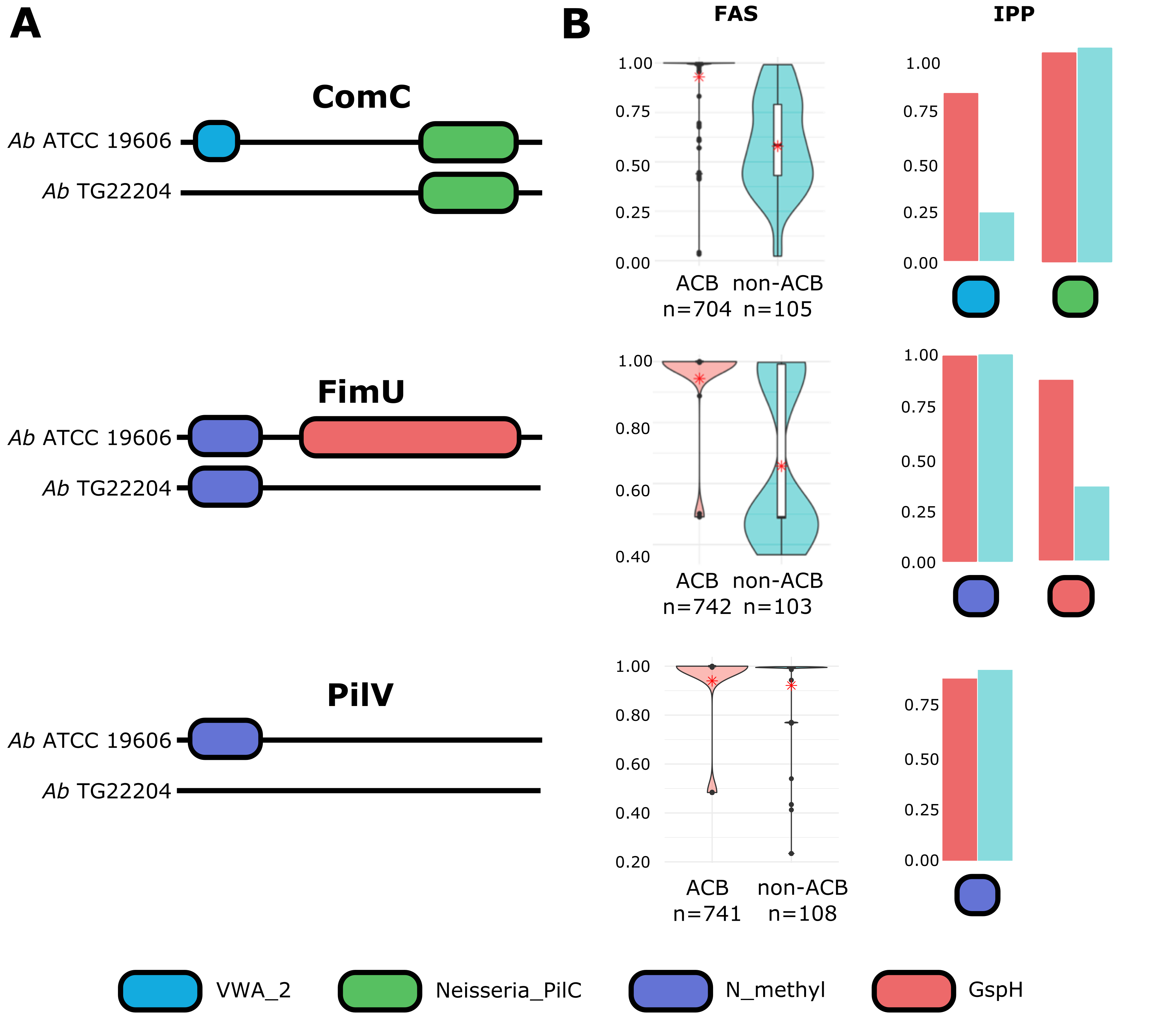

Supplement: S6 Fig — (A) The main domain architecture (DA) variants of ComC, FimU, and PilV respectively, in the genus Acinetobacter. (B) Feature architecture similarity (FAS) scores between each of the three Ab ATCC 19606T proteins shown in (A) and their orthologs. The protein of Ab ATCC 19606T is used as reference. FAS scores range from a maximum of 1 (identical architectures) to 0 (no shared domains) [45]. FAS score distributions are given separately for isolates inside (red) and outside (blue) of the ACB complex. The asterisk marks the FAS score mean. The average number of instances per protein (IPP) for the individual domains is represented by the bar plot. Pfam domains: VWA_2—PF13519; Neisseria_Pil_C—PF05567; N_Methyl—PF07963; GspH—PF12019. (PNG) [file pgen.1010646.s006.png]

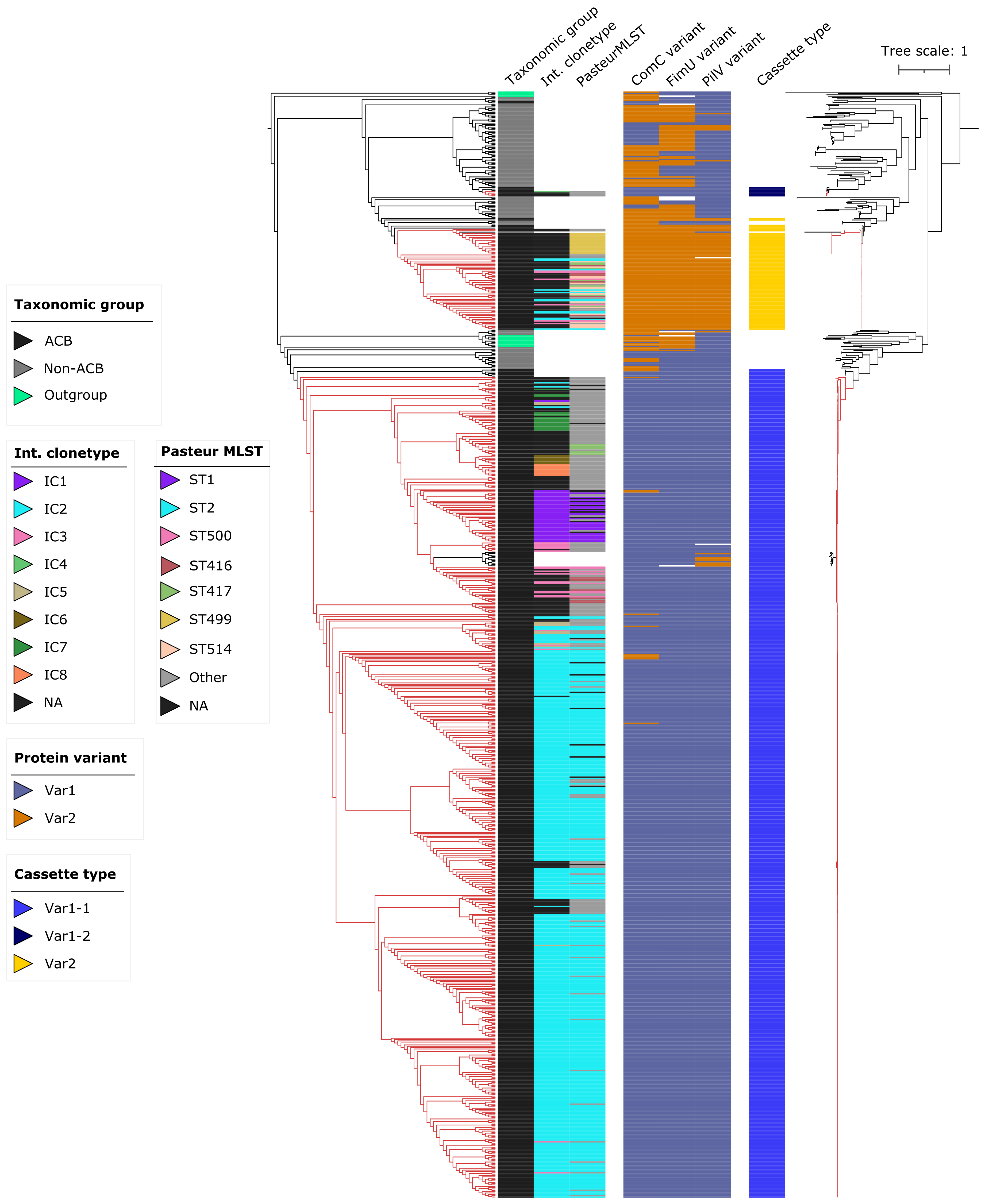

Supplement: S7 Fig — The evolutionary relationships of the ComC orthologs are reflected in the left cladogram. A. baumannii isolates are indicated by red branches, isolates of all other species are indicated by black branches. The tree including branch lengths is provided to the right of the figure. Annotations show from left to right for each Acinetobacter isolate the taxonomic group assignment, the international clonetype, and the strain type according to the Pasteur scheme. Sequence types according to the Pasteur scheme are specified only when more than 10 isolates share the same strain types. Otherwise, they are summarized under ‘other’. The next annotation columns provide for each bacterial isolate the protein variants of ComC, FimU and PilV, and the cassette type. A high-resolution version of this tree is available via figshare: https://figshare.com/articles/figure/_/21967694. (PNG) [file pgen.1010646.s007.png]

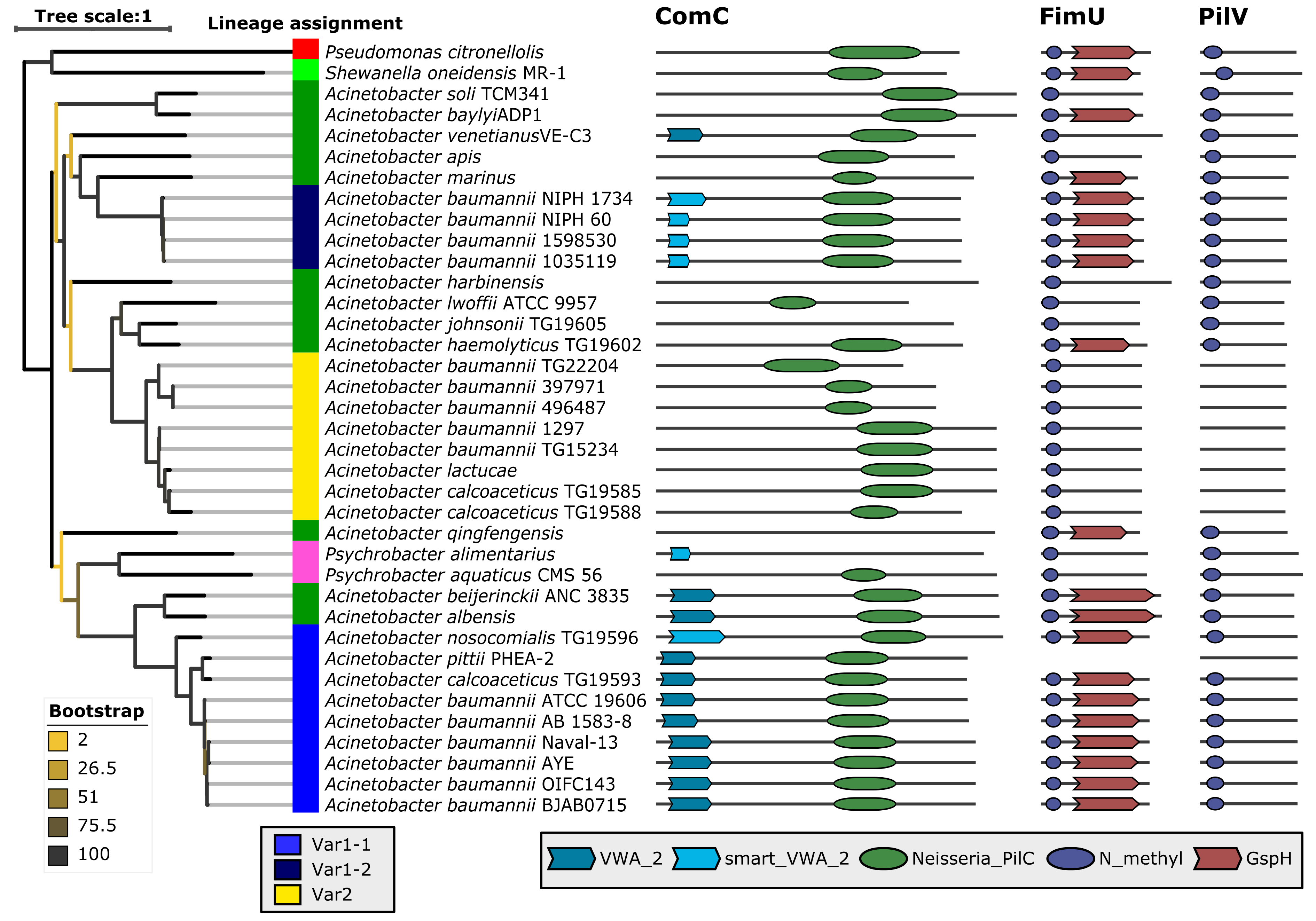

Supplement: S8 Fig — The tree shows a selection of taxa from the full set that represents the diversity of domain architectures of ComC, FimU, and PilV. The color code of the leaf labels resembles that of Fig 3 in the main text. The Pfam domain architectures of the three proteins in the represented isolates are given next to the taxon names. (PNG) [file pgen.1010646.s008.png]

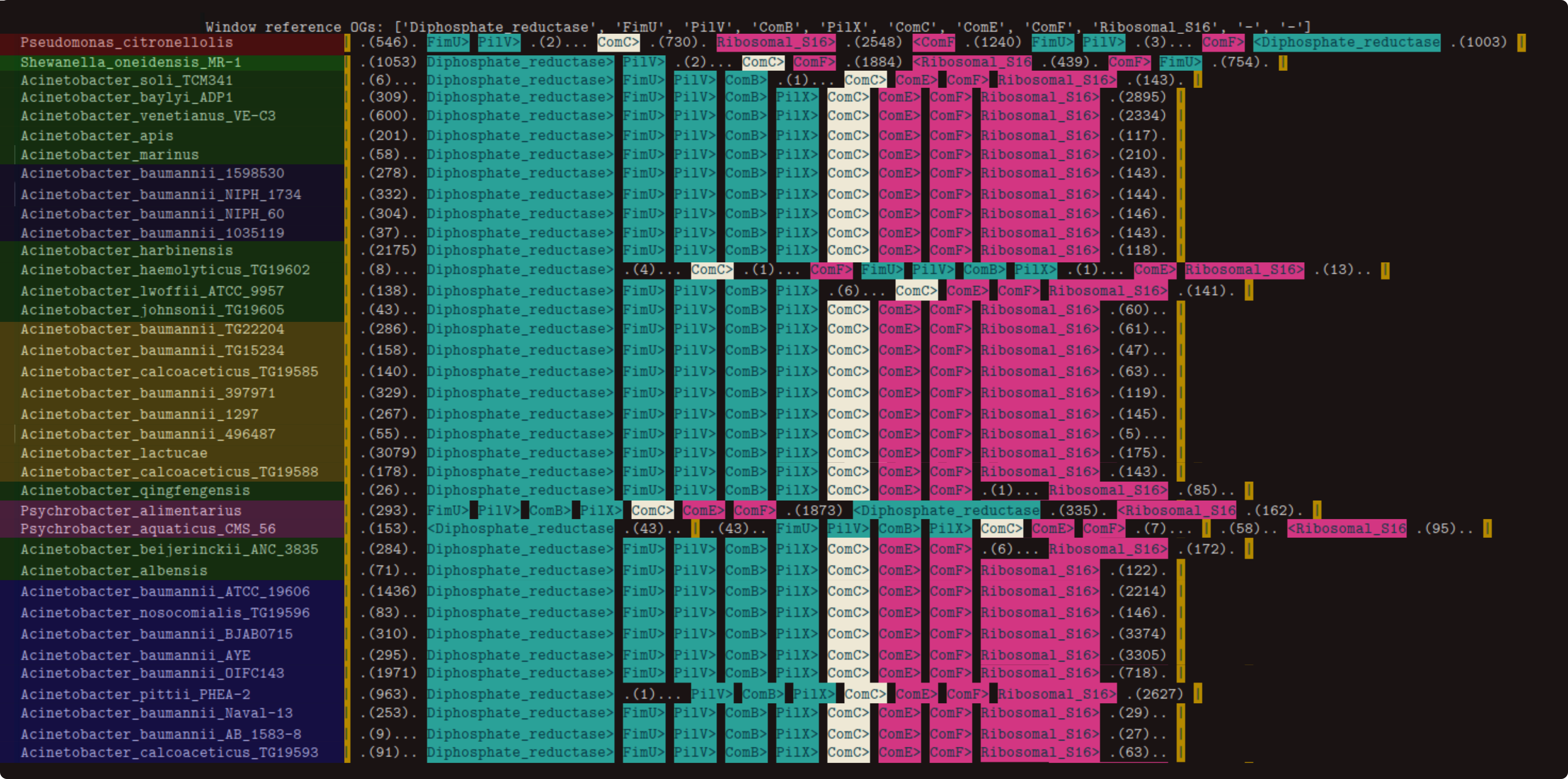

Supplement: S9 Fig — Each line in the plot represents the bacterial isolate that is indicated to the left. The color coding of the taxon labels corresponds to their lineage assignment in Fig 3 of the main text. Each box represents an ortholog to one of the 13 genes in the ComC/FimU region of Ab ATCC 19606T (see main Fig 3) and the gene identity is given by the box label. The order of boxes corresponds to the order of genes in the genome of the respective isolate. comC orthologs are marked in white. Green and red boxes identify genes that are upstream and downstream of comC in Ab ATCC 19606T, respectively. The number of genes separating any two of the boxed genes or that are placed upstream or downstream up to the scaffold end (indicated by yellow bars) are given in parenthesis. The plot confirms that the gene order in this region is conserved throughout the genus with very few exceptions. (PNG) [file pgen.1010646.s009.png]

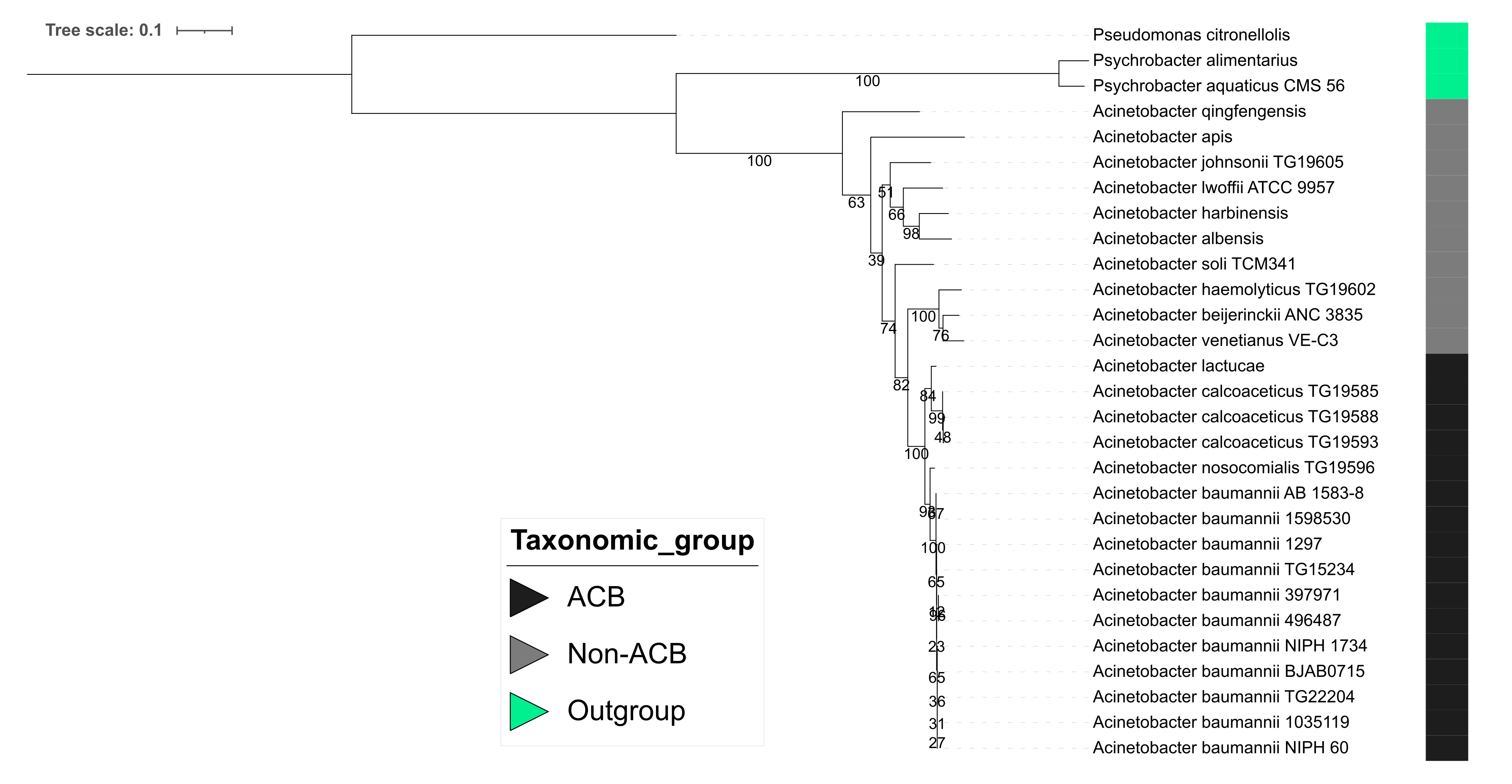

Supplement: S10 Fig — The gene tree is based on a multiple amino acid sequence alignment of the genes flanking the T4aP components in Fig 3 of the main manuscript. It comprises the following proteins: guanylate kinase (WP_000015937.1), 4-hydroxy-3-methylbut-2-enyl diphosphate reductase (WP_000407064.1), 30S ribosomal protein S16 (WP_000260334.1), ribosome maturation factor RimM (WP_000189236.1), tRNA (guanosine(37)-N1)-methyltransferase TrmD (WP_000464598.1), and 50S ribosomal protein L19 (WP_000014562.1). NCBI accession numbers are given in parenthesis. Branch labels denote percent bootstrap support. (PNG) [file pgen.1010646.s010.png]

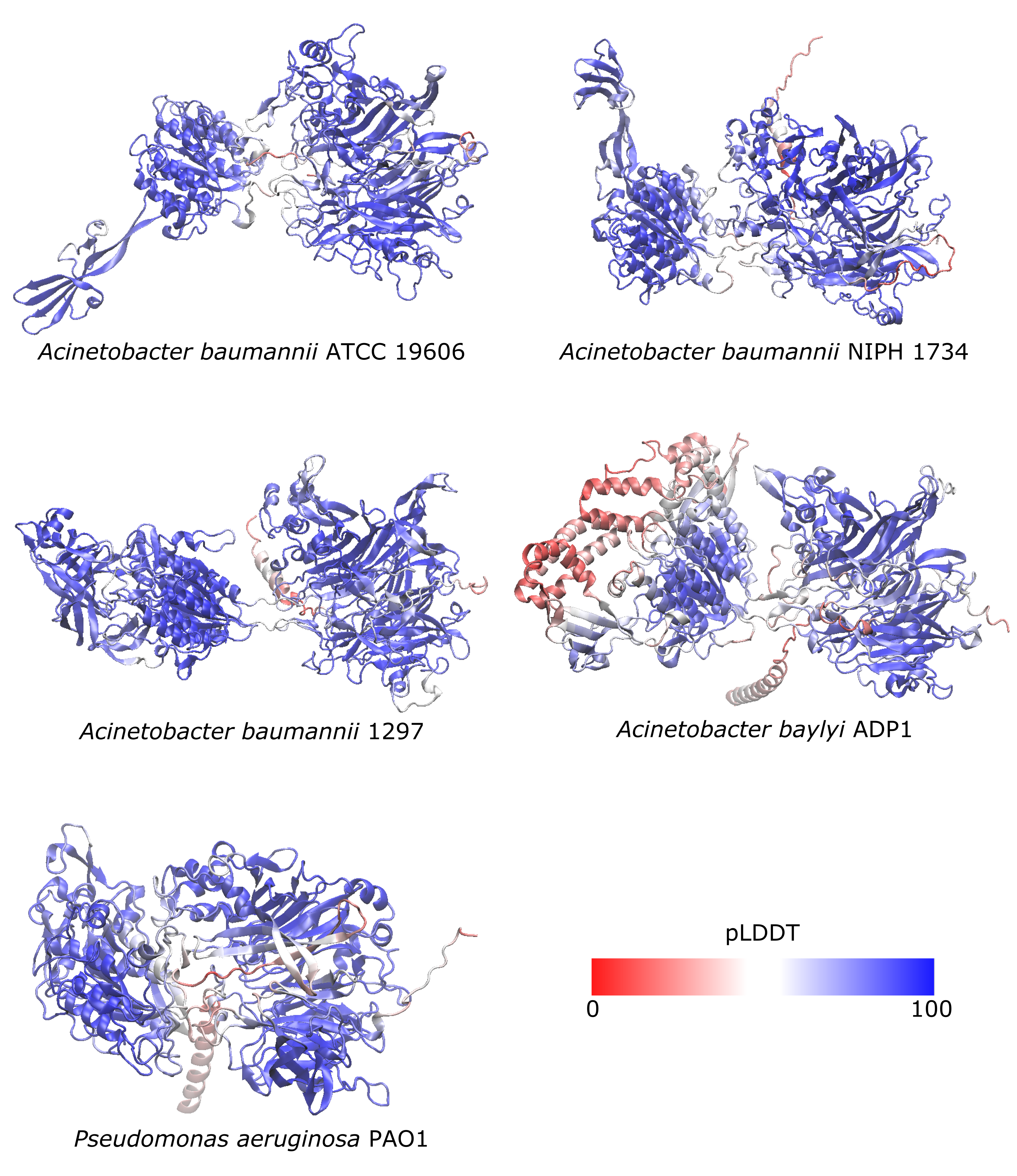

Supplement: S11 Fig — The color coding in the structures represents the per-residue confidence scores (pLDDT) provided by AlphaFold2 [114]. All structures are arranged such that the N-terminal domain is oriented to the left and the C-terminal domain to the right. (PNG) [file pgen.1010646.s011.png]

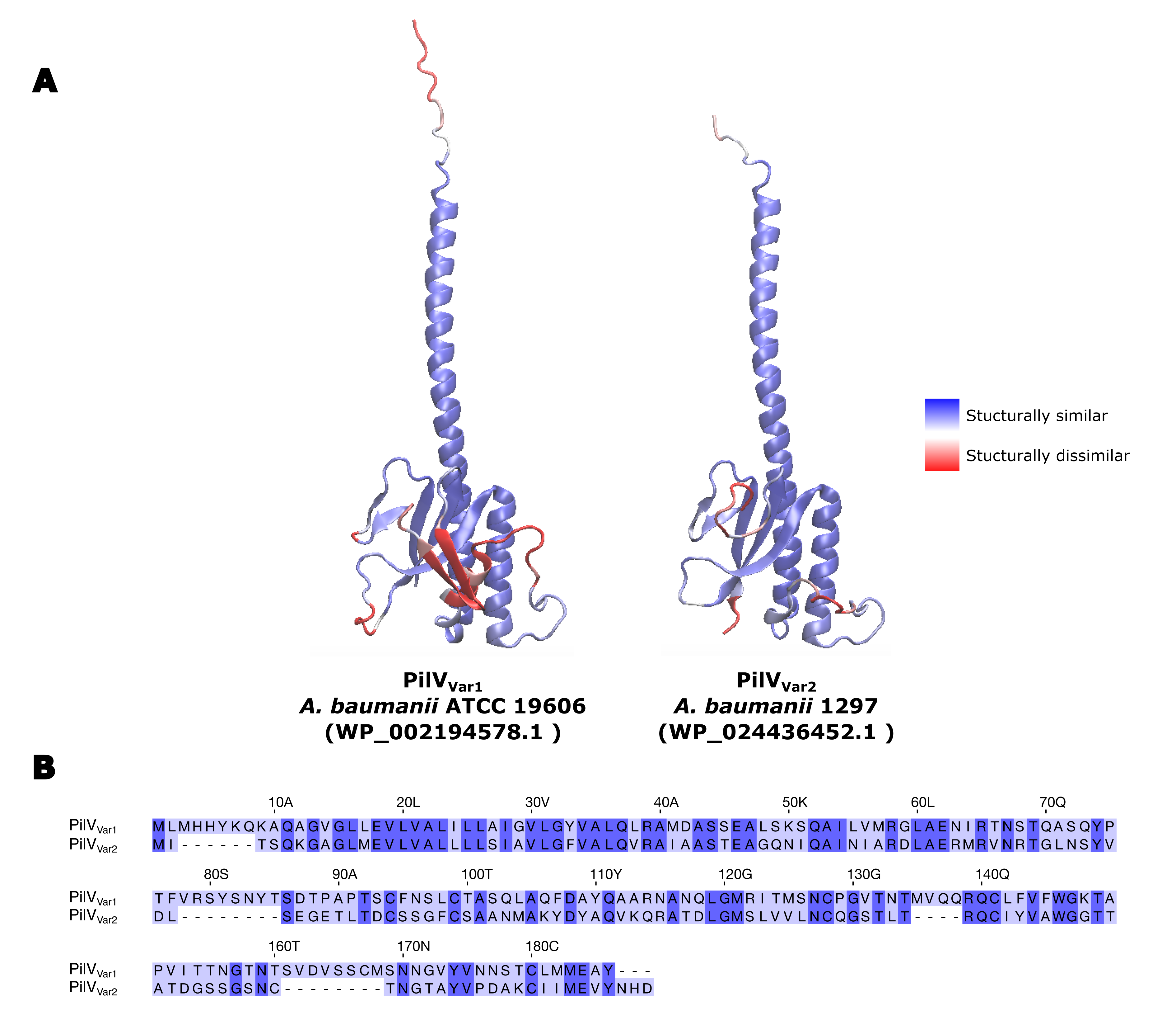

Supplement: S12 Fig — (A) Comparison of the modelled 3D structures of PilVVar1 (A. baumannii ATCC 19606T) and PilVVar2 (A. baumannii 1297). The structures are colored according to their mutual structural similarity from high (blue) to low (red). (B) Pairwise sequence alignment of the two PilV variants. Residue numbering refers to PilV in Ab ATCC 19606T. The non-detection of the N-terminal methylation motif (PF07963) in PilVVar2 is due to the substitutions at positions 14 (V->A) and 17 (L->M) in the alignment. (PNG) [file pgen.1010646.s012.png]

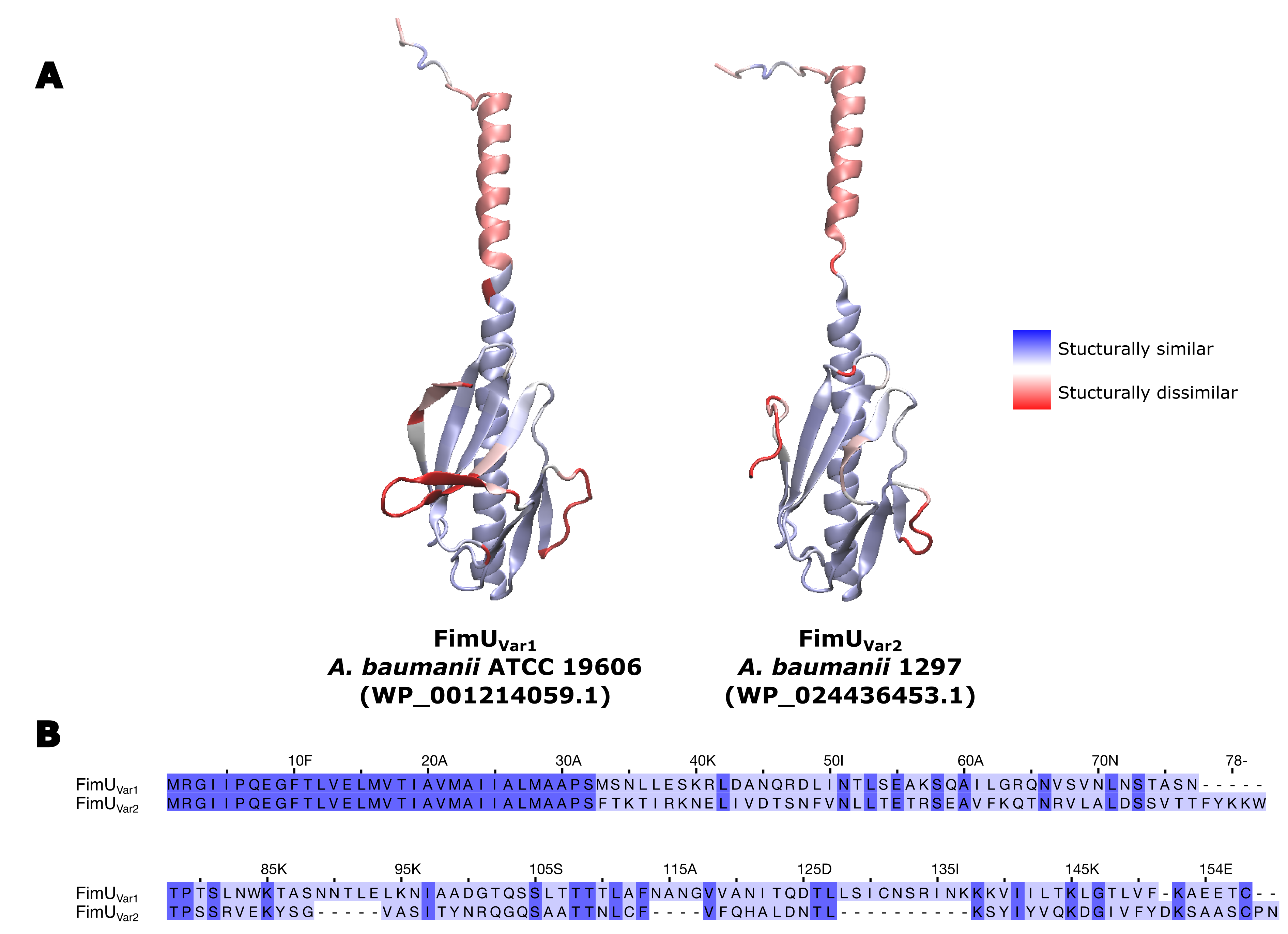

Supplement: S13 Fig — (A) Comparison of the modelled 3D structures of FimUVar1 (A. baumannii ATCC 19606T) and FimUVar2 (A. baumannii 1297). The structures are colored according to their mutual structural similarity from high (blue) to low (red). Red colored alpha helices in both structures correspond to the signal peptide. (B) Pairwise sequence alignment of the two FimU variants. Residue numbering refers to FimU in Ab ATCC 19606. (PNG) [file pgen.1010646.s013.png]

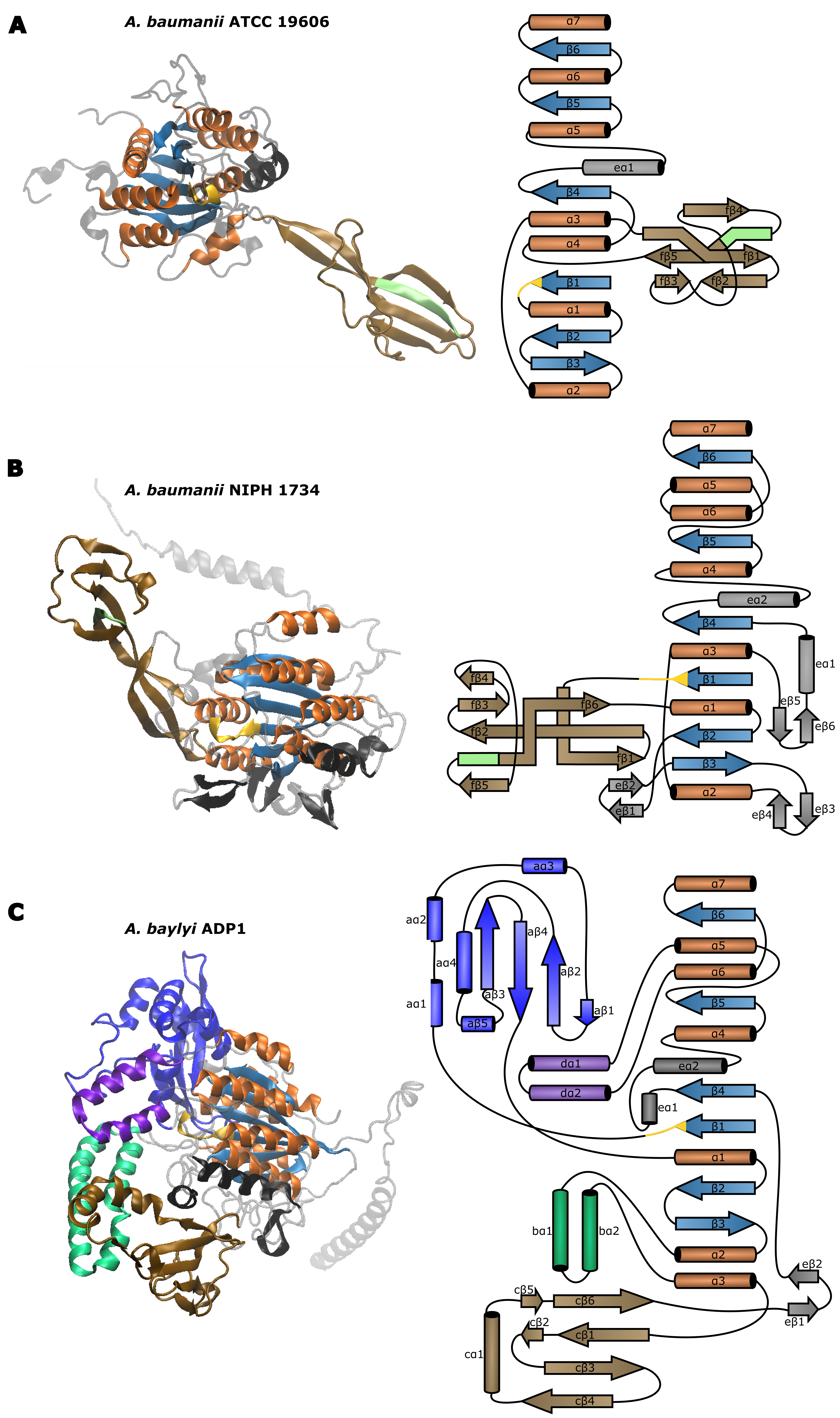

Supplement: S14 Fig — Alpha helices (orange) and beta strands (blue) are shown in the 3D cartoon representation (left) and in the secondary structure topology plots of sample proteins (right). Highlighted are the MIDAS motif (yellow), tyrosine-rich motif (light green), subdomains (brown and bright blue), and external folds (grey, purple, dark green). A. baumannii ATCC 19606T (A) and A. baumannii NIPH 1734 (B) feature an antiparallel beta sheet finger-like protrusion (brown). (C) The structure of A. baylyi contains additional folds (dark blue, purple, green, and brown) that are highly disordered and form a planar shield that surrounds the MIDAS motif. (PNG) [file pgen.1010646.s014.png]

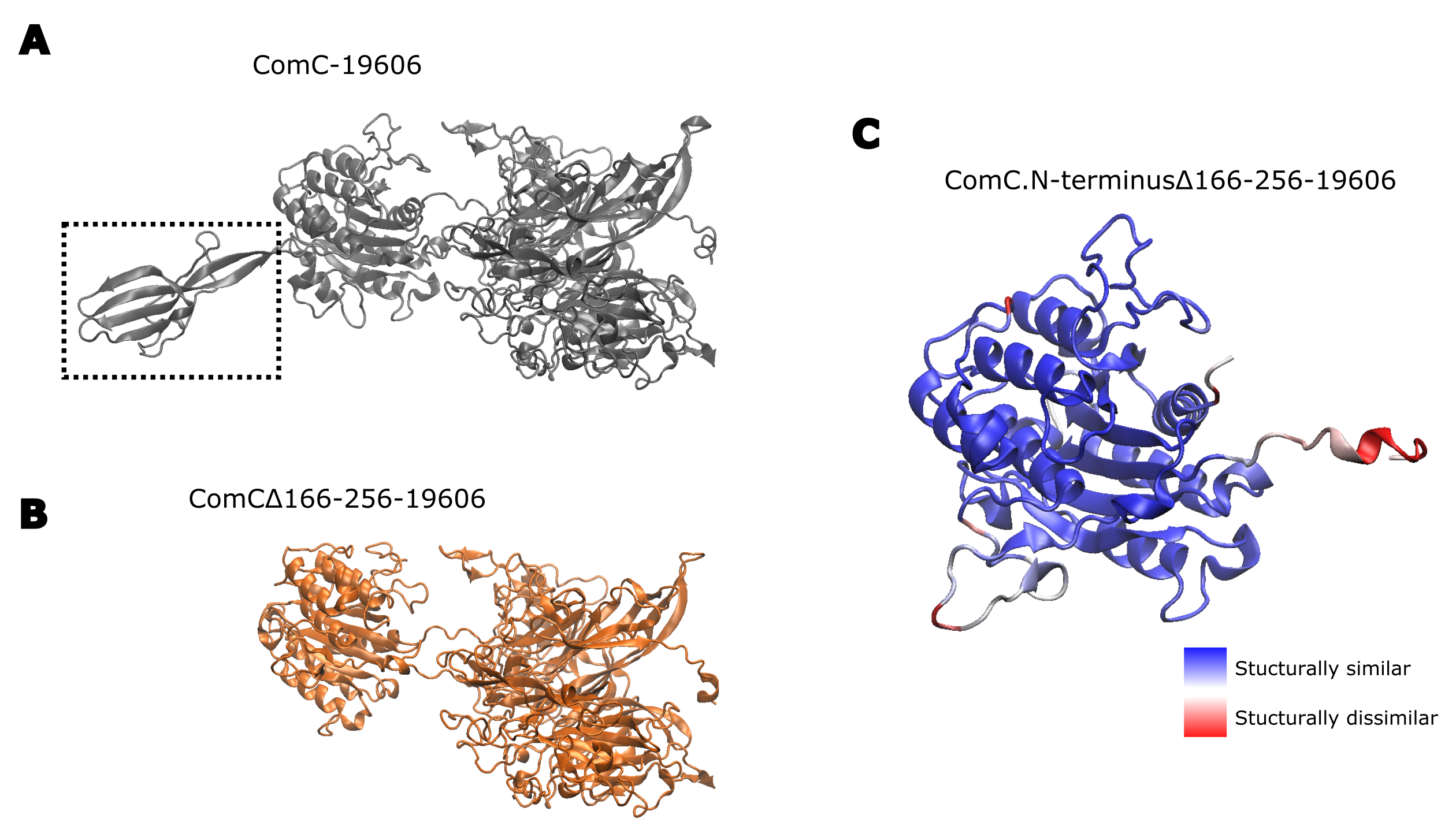

Supplement: S16 Fig — The predicted structures of Ab ATCC 19606T ComC and of the mutant lacking the finger like protrusion are shown in panels (A) and (B), respectively. The confidence in the predicted structure for the mutant lacking the finger-like protrusion is high (pLDDT = 90.06) [114]. The hatched box in (A) indicates the finger in the wild-type protein. A structural alignment of the two N-terminal globular domains is shown in (C), where structurally conserved regions are shown in blue. The pLDDT of the mutant is 90.06. (PNG) [file pgen.1010646.s016.png]
