# Supplementary material for: Feature architecture aware phylogenetic profiling indicates a functional diversification of type IVa pili in the nosocomial pathogen Acinetobacter baumannii
Source: PLoS Genet. 2023 Jul 27;19(7):e1010646. doi: 10.1371/journal.pgen.1010646 (PMC10374093; doi:10.1371/journal.pgen.1010646)
Supplement: Attachment — Submitted filename: ComC-AR.docx [file pgen.1010646.s023.docx]

## **Revision ComC Manuscript - Answer to the reviewers’ comments**

Before we address the individual points raised by the three reviewers, we would like to thank them for taking the time to critically assess our manuscript, and for the very careful and constructive review. We have organized our response in the following way.

1. We first provide the *reviewer comment in italics using blue font color*. To allow a better tracking, we have enumerated the individual comments, where Major Issues are identified by a capital letter ‘M’ before the number, whereas minor issues are identified by a ‘MI’
2. You will find our response directly below the comment in normal font (black)
3. In case, we changed or added text in the manuscript when following the reviewers’ suggestion, we provide the new text below our answer starting with ‘We write now:’ *followed by the new text in italics (red).*
4. If we modified an already existing figure, the new version is provided in this document. If we have added an analysis, which was performed to address the reviewers’ comments, to the manuscript’s online supporting information, then we have indicated this. **All figure numbers reflect those in the revised version of our manuscript.** If we added a figure to this response letter to support our answer to the reviewers, which is not used in the manuscript, then we refer to these figures as “Figure AR” followed by a number.
5. In individual cases, the **reviewers made largely overlapping comments**. To keep this answer letter concise, we only outline the answer the second time an issue was raised, and then refer to the already given answer. To simplify the navigation through the text, we provide interactive links that allow a direct navigation to the relevant information.

Please note, we provide no text if we amended the writing style or corrected typos or incorrect punctuation.

We hope that this structure helps to streamline the re-review.

Sincerely,

Ingo Ebersberger (on behalf of all authors)

[**Revision ComC Manuscript - Answer to the reviewers’ comments**	1](#_Toc136002024)

[Reviewer 1	5](#_Toc136002025)

[M1.1 - Fig 3 International clonetypes of A. baumannii genomes. I have a major concern regarding assignment of A. baumannii genomes reported in Fig. 3.	5](#_Toc136002026)

[MI1.1 - Introduction, lines 66-67: Acinetobacter baumannii is a Gram negative nosocomial pathogen that accounts for about 5% of the total bacterial infections worldwide [1-3].	7](#_Toc136002027)

[MI1.2 - Table 1. To avoid confusion, comP and pilA synonyms of pilin should be indicated both in the first column of the table as comP/ pilA instead of in the footnote.	7](#_Toc136002028)

[MI1.3 - Figure 1B and Figures 2 and 3. Inclusion of Acinetobacter oleivorans into Acinetobacter baumannii-calcoaceticus complex.	7](#_Toc136002029)

[MI1.4 - Also, I invite Authors to correct international clonal type assignment of Figure 3.	9](#_Toc136002030)

[MI1.5 - Domain architecture difference between FimT orthologs reported in Results section on lines 153-155.	10](#_Toc136002031)

[MI1.6 - Discussion, lines 350-362.Differences between ACICU and ATCC19606 PilA variants and comC – FimU genes should be explained…	11](#_Toc136002032)

[MI1.7 - Reference nr. 20. Please, correct title.	11](#_Toc136002033)

[Reviewer 2	11](#_Toc136002034)

[M2.1 - The only significant concern I have with the interpretation of the data is how to interpret the loss of T4P function in the comCΔVWA and comCΔ166-256 mutants.	11](#_Toc136002035)

[MI2.1 - Based on figures 1 and 3, it appears that pilV has similar levels of variation and may be covariant with comC and fimU…	13](#_Toc136002036)

[MI2.2 It is no fault of the authors because the naming of T4P genes and gene-products in the literature is a mess.	17](#_Toc136002037)

[MI2.3 In Figure 1C, I am concerned that the figure overstates the differences between some of these genes.	18](#_Toc136002038)

[MI2.4 - In Figure 4 (panels A and B), it is difficult to follow what is happening with the superimpositions at this figure resolution	19](#_Toc136002039)

[Reviewer 3	22](#_Toc136002040)

[General remarks of the authors	22](#_Toc136002041)

[M3.1 - Line 131. “Orthologs to all”... Please start the paragraph saying a word on the methodology for "ortholog search", otherwise the entire paragraph is hard to follow.	24](#_Toc136002042)

[M3.2 - Line 152-153: rather than “chance effects” that sounds a bit odd, couldn’t the authors rather involve variations in sequences…	25](#_Toc136002043)

[M3.3 - On line 390 of the Methods, it would be very important to indicate what parameters were used for HMMER?...	26](#_Toc136002044)

[M3.4 - We touch here the heart of my main criticism:	26](#_Toc136002045)

[M3.4.1 - I do not understand how domains were annotated in details…	26](#_Toc136002046)

[M3.4.2 - For example on lines 156-158 the authors then claim themselves “The domain loss in the A. baumannii type strain is caused by a substitution…	27](#_Toc136002047)

[M3.4.3 - Again, what is unclear to me here and later on, is whether the domain is really “lost” or gained…	28](#_Toc136002048)

[M3.4.4 - It probably does not change the functional interpretation, this domain diversification might be as well very functionally relevant…	29](#_Toc136002049)

[M3.5 - On lines 166, it is indicated that “We next investigated the evolutionary histories of ComC and FimU in greater detail.”…	29](#_Toc136002050)

[M3.6 - Fig. 2 represents a Maximum-Likelihood phylogeny, but neither branch lengths nor branch supports appear…	30](#_Toc136002051)

[M3.7 - A recombination analysis followed, but it is difficult to grasp how its results articulate with that of the domain analyses:	30](#_Toc136002052)

[M3.8 - Regarding the recombination analysis, the paragraph should contain a word on which principle was used for recombination detection…	31](#_Toc136002053)

[M3.9 - Line 216-218: “Therefore, the two fingers are very likely of different evolutionary origins…	32](#_Toc136002054)

[M3.10 - I am not a structural biologist, but what is the confidence of the 3D models obtained with AlphaFold in the precise regions of interest for ComC on Fig 4A?...	33](#_Toc136002055)

[M3.11 - The proposed mutant comC to test experimentally are based on mutant proteins that seem somehow drastic versions of it….	34](#_Toc136002056)

[M3.12 - I am not convinced that the experimental results found in Fig. 5 demonstrate specifically the role of the VWA_2 PFAM (nor or the finger) domain in host cell adhesion, twitching motility or competence…	34](#_Toc136002057)

[MI3.1 - Line 36, abstract section: “Both genes together form an evolutionary cassette” is not totally reflecting the scenario proposed by the authors…	35](#_Toc136002058)

[MI3.2 - I found the end of introduction too detailed on the results, it is somehow redundant with an abstract.	35](#_Toc136002059)

[MI3.3 - I do not see the connection with the paragraph on lines 124-137 and the very 1st line of the paragraph…	36](#_Toc136002060)

[MI3.4 - Fig. 1B. it is unclear what should be compared here. Also, I do not get the meaning of the FAS_R score?...	36](#_Toc136002061)

[MI3.5 - Lines 149-150: “the architectures of four proteins, PilX, FimT, FimU, and ComC deviate to an extent between orthologs that it could indicate a change in function.”…	36](#_Toc136002062)

[MI3.6 - Line 124: Included in what? Please rephrase as it is unclear.	37](#_Toc136002063)

[MI3.7 - Line 170-171, it is mentioned that ComC and FimU are close in the genomes of Ab ATCC 19606 T is this also the case in other Acinetobacter genomes?	38](#_Toc136002064)

[MI3.8 - Figure 2: what are the black branches?	38](#_Toc136002065)

[MI3.9 - Line 185-186: “This indicates that recombination has affected the evolution of this locus”…	38](#_Toc136002066)

[MI3.10 - Line 386: "RefSeq database release 204" Could the authors mention on what date it was last accessed?	38](#_Toc136002067)

[Typos	38](#_Toc136002068)

[Typo1 - Line 74 : remove «how »	38](#_Toc136002069)

[Typo2 - Line324: PDB means “Protein Data Bank”. Authors may want to add in text it is a database of structures of proteins?	39](#_Toc136002070)

[Typo3 - Line 413: where => were	39](#_Toc136002071)

[References	40](#_Toc136002072)

## Reviewer 1

*Reviewer #1: Manuscript PGENETICS-D-23-00119 is an interesting and well written study on the phylogenetic profiling of type IVa pili in Acinetobacter baumannii. The manuscript adds novel information on the mechanisms of adhesion, twitching motility and natural competence in Acinetobacter and is of interest for the readers of the journal. I am listing below specific queries which need to be addressed by Authors.*

### M1.1 - Fig 3 International clonetypes of A. baumannii genomes. I have a major concern regarding assignment of A. baumannii genomes reported in Fig. 3.

*It has been shown by several publications that A. baumannii has a clonal population structure dominated by three global/international clonal lineages, named GC1, 2 and 3, and few additional epidemic clonal lineages, which emerged worldwide (Zarrilli et al. Int.J. Antimicro Agents. 2013, 41, 11-19). Among several typing methods used, it has been demonstrated that the most appropriate to study A. baumannii genomes and assign to epidemic clonal lineages is PasteurMLST-based typing scheme (Gaiarsa et al. Front. Microbiol. 10:930, 2019). I invite Authors to assign A. baumannii genomes included in Figure 3 and in Supplementary Table 3 to international clonal types using Pasteur MLST scheme as described in Gaiarsa et al. Front. Microbiol. 10:930, 2019 and taking advantage of additional information found in AcinetobacterPubMLST genomes databases at* [*https://pubmlst.org/bigsdb?db=pubmlst_abaumannii_isolates&page=query&genomes=1*](https://pubmlst.org/bigsdb?db=pubmlst_abaumannii_isolates&page=query&genomes=1)

Thank you very much for this thorough summary of *A. baumannii* strain typing. We used the 8 international clone types assigned by Higgins and colleagues [1] already in Djahanschiri et al. (2022), of which our data set is a subset from. We therefore adopted the typing scheme here for consistency reasons. In Djahanschiri et al. (2022) we also performed strain typing according to the Pasteur- and Oxford schemes. However, the strain typing itself provides no additional insights into the evolution of the T4aP cluster. **Moreover, we find it hard to accommodate 14 different Pasteur sequence types in Fig. 3**. We therefore prefer to stay with the international clonetypes in Fig 3. However, now **we integrate the information about cassette type, international clonetype, and Pasteur sequence type in the updated S7 Figure (see below).** Moreover, the alternative sequence typing according to the Pasteur scheme is provided in S3 Table and in S7 Figure. In the caption of Fig. 3 we write now:

*(…) The taxonomic assignments together with the international clonetype (ICs) assignments for the A. baumannii isolates [1] are indicated by the colored boxes left of the taxon labels. Multilocus sequence typing of the A. baumannii isolates according to the Pasteur scheme is provided in S3 Table and S7 Figure. (…)*

*S7 Figure. Maximum likelihood phylogeny of ComC.* *The evolutionary relationships of the ComC orthologs are reflected in the left cladogram. A. baumannii isolates are indicated by red branches, isolates of all other species are indicated by black branches. The tree including branch lengths is provided to the right of the figure.* *Annotations show from left to right for each Acinetobacter isolate the taxonomic group assignment, the international clonetype, and the strain type according to the Pasteur scheme. Sequence types according to the Pasteur scheme are specified only when more than 10 isolates share the same strain types. Otherwise, they are summarized under ‘other’. The next annotation columns provide for each bacterial isolate the protein variants of ComC, FimU and PilV, and the cassette type. A high-resolution version of this tree is available via figshare: https://figshare.com/articles/figure/_/21967694*

**To conclude this part**, the tree shown in Fig. 3 serves the purpose of showing that the exchange of the T4aP component cassette post-dates the diversification of *A. baumannii* into contemporary strains. We added the strain typing only to show that the pilus variants we find are not limited to a particular international clonetype.

### MI1.1 - Introduction, lines 66-67: Acinetobacter baumannii is a Gram negative nosocomial pathogen that accounts for about 5% of the total bacterial infections worldwide [1-3].

*Authors need to reconsider the statement according to more recent evidence of the literature. I suggest the following three recent publications: Nowak, J. et al. (2017) J. Antimicrob. Chemother. 72, 3277–3282; Wong et al. 2017. Clin Microbiol Rev 30:409–447; Tacconelli et al. 2018. Lancet Infect Dis 18:318–327.*

Thank you very much for this input. We revisited this issue and use now a study that was published 2022 in Lancet (Murray CJL, et al. Global burden of bacterial antimicrobial resistance in 2019: a systematic analysis. Lancet. 2022;399(10325):629-55.) to back up the statement that *A. baumannii* is a serious threat to human health. **We write now:**

*Acinetobacter baumannii is a Gram negative nosocomial pathogen. A recent world-wide survey estimated that antimicrobial resistant A. baumannii strains were responsible for 10.4% of bacterial infections with fatal outcome in 2019 [2].*

### MI1.2 - Table 1. To avoid confusion, comP and pilA synonyms of pilin should be indicated both in the first column of the table as comP/ pilA instead of in the footnote.

The issue of name equivalences was also pointed out by reviewer 2. We have adapted Table 1 to include the names of orthologous proteins in *P. aeruginosa*. Pleae see also our answer to MI2.2

### MI1.3 - Figure 1B and Figures 2 and 3. Inclusion of Acinetobacter oleivorans into Acinetobacter baumannii-calcoaceticus complex.

*Authors should indicate the methods/criteria used to include A. oleivorans into A. baumannii-calcoaceticus complex. While it is evident that A. olevoirans DR1 isolates is a distinct species of Acinetobacter genus (Kang et al. J Microbiol. 2011;49(1):29-34), I did not find any A. oleivorans isolate identified at species level using MALDI-TOF (Marí-Almirall et al. Clin Microbiol Infect. 2017; 23: 210.e1-210.e9) or Raman Spectrometry (Molecules 2019, 24, 168). Instead, it is possible to perform species identification of A. oleivorans genomes using ribosomal MLST (Jolley et al. 2012; Microbiology 158:1005-15). A collection of genomes assigned to 72 distinct species into Acinetobacter genus is available at in AcinetobacterPubMLST genomes databases at*

[*https://pubmlst.org/bigsdb?db=pubmlst_abaumannii_isolates&page=query&genomes=1*](https://pubmlst.org/bigsdb?db=pubmlst_abaumannii_isolates&page=query&genomes=1)

*If identification at species level of Acinetobacter genomes included in the manuscript has been performed using genome sequences and/or ribosomalMLST allele profiles, I suggest to modify genomes classification by grouping A. baumannii, A. nosocomialis, A. pittii, A. seifertii and A. lactucae genomes into A. baumannii group and all other genomes into non-baumannii Acinetobacter group. This approach has been adopted using MALDI-TOF identification for Acinetobacter spp. (Marí-Almirall et al. Clin Microbiol Infect. 2017; 23: 210.e1-210.e9).*

Thank you very much for pointing out that *A. oleiovorans* is a tricky taxon, and we very much appreciate the very detailed information provided by the reviewer. **Our assignment of the *A. oleivorans* isolates to the ACB clade is phylogenetically motivated**. In Tietgen et al. (2019) [3], we investigated the evolutionary relationships of *A. calcoaceticus*, *A. oleivorans*, and *A. baumannii* based on a phylogenomic dataset comprising more than 2,000 1:1 orthologs. This revealed that the three *A. oleivorans* isolates are nested within the *A. calcoaceticus* diversity (Fig. AR1). In Djahanschiri et al. (2022) [4], we performed a phylogenomic reconstruction of the evolutionary relationships covering the entire *Acinetobacter* diversity, which was known by that time. We found again that the *A. oleivorans* isolates are grouping together with A. calcoaceticus and consistently within the diversity of the ACB clade (Fig. AR2).

The reviewer suggests summarizing *A. baumannii*, *A. nosocomialis*, *A. pittii*, *A. seifertii*, and *A. lactucae* into the “*A. baumannii* group”, and the remainder of the species/genomes into the “non-baumannii group”. We trust that there are reasons to do so, however they are not evolutionary motivated, which is our approach here. In Djahanschiri et al. 2022 [4] we have unambiguously shown that ***A. calcoaceticus* and the *A. oleivorans* isolates share a common ancestor with *A. pittii* and *A. dijkshoorniae*** **that existed after the evolutionary lineages leading to contemporary *A. baumannii*, *A. nosocomialis* and *A. seifertii* have branched off.**

Figure AR1. Maximum likelihood phylogeny of A. baumannii, A. calcoaceticus and A. oleivorans. The tree shows that the three A. oleivorans isolates fall within the diversity of A. calcoaceticus. This tree is Fig. 1 from [3].

*Figure AR2. Consistency based Maximum likelihood phylogeny of 232 Acinetobacter isolates representing the diversity of the genus as it was known by the time of the analysis. The tree is the consensus of three non-overlapping phylogenomic data sets, each comprising ~290 Acinetobacter core genes. A. oleivorans is unambiguously placed as a sister clade to A. calcoaceticus. This tree is Fig. 2A from [4]. Fig. S10 from this publication provides a tree covering more than 3,000 Acinetobacter isolates, and again it supports the placement of the A. oleivorans isolates within the ACB clade (https://doi.org/10.1371/journal.pgen.1010020.s011).*

To conclude our answer to this issue, **we use a phylogenetic approach to assign members to the ACB clade. Phylogenetic evidence is unambiguous in placing the investigated *A. oleivorans* isolates within the diversity of the ACB clade.** What could be questioned is whether *A. oleivorans* is a valid species. However, this is not relevant for our findings, and clarifying this issue is therefore beyond the scope of this study. With respect to the criteria used to order taxa in our phylogenetic profile, we wrote in the Methods subsection *Domain-architecture-aware ortholog search:* “*The taxa represented in the phylogenetic profiles were ordered with increasing phylogenetic distances from A. baumannii ATCC 19606 T using the Acinetobacter phylogeny in [21] and the γ−proteobacteria tree from [93].*”. We moved this information now to the caption of Figure 1. **We write now:**

*Figure 1. (…) (B) Phylogenetic profiles of the Ab ATCC 19606 ^T^ T4aP components across 884 bacterial isolates representing 83 species. Taxa are summarized on the species level. A dot indicates the presence of an ortholog in the respective species, and the dot size represents the fraction of the subsumed isolates an ortholog was identified in. The color encodes the median feature architecture similarity (FAS score) between the protein in Ab ATCC 19606 ^T^ and its orthologs within a species. The dot color gradient (FAS_F; blue to orange) captures architecture differences using the feature architecture of the Ab ATCC 19606^T^ protein as reference. The score decreases if features seen in the Ab ATCC 19606^T^ protein are missing in the respective orthologs. The cell color gradient (FAS_B; white to pink) captures architecture differences using the feature architecture of the ortholog as reference. The score decreases if features seen in the ortholog are missing in the Ab ATCC 19606^T^ protein. Taxa are ordered according to increasing phylogenetic distance to A. baumannii using the information provided in [4] and [5]. (…)*

### MI1.4 - Also, I invite Authors to correct international clonal type assignment of Figure 3.

*In fact, Acinetobacter baumannii TG215234 and Acinetobacter baumannii ATCC19606 are both assigned to international clonal type 2. This is true for Acinetobacter baumannii TG215234, while A. baumannii ATCC19606 has a distinct clonal type (assigned to PasteurST25 type). Moreover, I noticed that Authors considered in supplementary Table 3 an old version of A. baumannii ACICU genome sequence, while thay should consider the revised complete genome sequences of Acinetobacter baumannii strains AB307-0294 and ACICU belonging to global clones 1 and 2 published recently by Hamidian M et al. Insights from. Microb Genom. 2019 Oct;5(10):e000298.*

Thank you very much for spotting this. We have corrected the erroneous clone type assignment of *Ab* ATCC 10606 in Fig. 3 (see below) and in S6 Figure. We now also used the most recent genome assemblies for *Ab* ACICU and *Ab* 307-0294. As expected, this did not change any of our findings.

*Fig. 3. Patterns of ancestral recombination in the locus surrounding the comC gene*

### MI1.5 - Domain architecture difference between FimT orthologs reported in Results section on lines 153-155.

*Authors should discuss whether this might be responsible for the absence of twitching motility in A. baumannii ACTCC 19606 strains observed in this study and in previous ones.*

This is a very interesting aspect, and we had now a closer look into this matter. We investigated FimT of six twitching strains and of further five non-twitching strains [6]. This revealed that FimT of two non-twitching strains differ solely in the I->T substitution from the twitching stains. This substitution is altering the prepilin peptidase cleavage motif in FimT [7] suggesting that it is probably causal for the loss of twitching. The remaining three non-twitching strains displayed an intact cleavage site; however, their FimT differs from that of the twitching strains by an R->H substitution at position 91 in the FimT alignment. We summarize this analysis now in S5 Figure and now briefly mention this in the Results section. **We write now:**

*The situation is different for FimT. Here, the feature architecture differences are caused by the sporadic non-detection of a N-terminal methylation motif, for example in FimT_Ab ATCC 19606_ (PF079631; Figs 1D and S4 Fig A-B). In this protein, the absence of PF079631 from the feature architecture coincides with a threonine at a position in the amino acid sequence that is typically occupied by a leucine or an isoleucine in PF07963 (S4 Fig C-E). This position is part of a prepilin peptidase cleavage motif ([7]; S5 Fig), and interestingly the change to a threonine is one of the two substitutions that characterizes A. baumannii strains that lost the ability to twitch ([6]; S5 Fig). Thus, there is evidence that functionally different variants of FimT exist in A. baumannii, however they are rare.*

*S5 Figure Comparison of FimT between twitching and non-twitching A. baumannii strains. Twitching and non-twitching A. baumannii strains were taken from [6] and the corresponging FimT proteins were aligned. The multiple sequence alignment shows only the parts of the FimT alignment that differ between the investigated strains. The color code represents the degree of sequence conservation. The five non-twitching strains are characterized by two substitutions in FimT. Two strains, including Ab ATCC 19606^T^, display an I->T substitution at position 9 in the alignment. This position is part of a prepilin peptidase cleavage motif [7] indicated in green above the alignment. The consensus motif together with the corresponding part of the N-methyl Pfam pHMM is shown on top of the green box (see also S4 Figure). The I->T substitution likely alters the function of the cleavage motif such that it interferes with the processing of FimT. As a consequence, the ability to twitch is lost. Three further non-twitching strains show the canonical prepilin peptidase cleavage motif, however they share an R->H substitution at Pos. 91 in the alignment. Because this is the only difference of these strains to the twitching A. baumannii strains, it is tempting to speculate that also the R->H substitution is sufficient to abolish twitching.*

### MI1.6 - Discussion, lines 350-362.Differences between ACICU and ATCC19606 PilA variants and comC – FimU genes should be explained…

*by differences in genetic background of ACICU and ATCC 19606 genomes.*

We would be more than happy to embed differences in the genetic background of *Ab* ACICU and *Ab* ATCC 19606 into the discussion. Unfortunately, we have no good idea what to write beyond what we already do. Our message is: PilA seems to structurally differ between the two strains, but they share the same layout of the ComC-FimU cassette. Thus, not only differences in the cassette layout, but also combinations of the same cassette layout with different PilA variants drive the diversity of T4aP in *Acinetobacter baumannii*. We would be happy to provide a more comprehensive analysis here, but unfortunately, we are limited to the few isolates considered in the study of Piepenbrink et al. [8]. Concerning any further explanation of our findings, we fear that we would become too speculative. We hope that the reviewer follows our view here.

### MI1.7 - Reference nr. 20. Please, correct title.

Thank you very much for spotting this, we have corrected the reference

## Reviewer 2

*Reviewer #2: This manuscript by Iruegas et al describes a pan-genomic comparison of type IV pilus (T4P) genes in the genus Acinetobacter. Based on this comparison, the authors found that for a specific cluster of those genes, recombination events had created substantial variation. In particular, the authors found the insertion of a VWA domain into comC, the tip adhesion, for some strains of A. baumannii, while others have the, apparently ancestral domain structure shared by A. baylyi. Alterations to this domain cause defects in multiple T4P functions in the A. baumannii AYE-T background.*

*This manuscript is well-written and contains new knowledge for those with an interest in the function and evolutionary origins of T4P systems. Previous analysis of variation in Acinetobacter T4P systems has been focused on the major subunit, pilA, while the fimU/comC variation appears to be the result of a separate, possibly earlier, evolutionary divergence.*

### M2.1 - The only significant concern I have with the interpretation of the data is how to interpret the loss of T4P function in the comCΔVWA and comCΔ166-256 mutants.

*While it is possible that the VWA structure plays a role in twitching motility, DNA-uptake and host-adherence, simpler explanation (particularly given the natural occurrence of functional non VWA ComC proteins) is that* ***ComC stabilizes the pilus structure and that the mutants are less stable than wild type ComC, leading to lower ComC expression and fewer pili***

We very much appreciate the overall very positive feedback of the reviewer. The main focus of our manuscript was the **application of a novel bioinformatics approach to scan evolutionary old proteins for evidences for a lineage specific change in function, which allowed us substantially broaden the scope of comparative sequence analyses between pathogenic and largely apathogenic *Acinetobacter* isolates**. Concerning the functional interpretation of our experimental work, we agree that we only started to investigate the role of the finger-like domain in *A.baumannii* ComC Var1.1, which is the most prominent difference between the various ComC-variants. As the reviewer has correctly pointed out, we cannot decisively rule out that the deletion of the finger-like domain affects the overall function of ComC to an extent that pilus formation and/or stability is impaired. Still, we trust that this is not the case for the following reasons:

1. Electron micrographs of the strain carrying the ComC-deletion mutant show **no indication of a substantial piliation defect** (see Fig. 5A).
2. Complementation with the **ComCΔ166-256 mutant at least partly restores natural competence** **but it does not rescue adhesion or twitching** (see Fig. 5B-D). Since all three functions likely depend on functional T4aP, this indicates that the phenotypic effects we see are not simple piliation defect.
3. Motivated by the reviewer’s critique, we have now **extended the experimental analysis and have complemented the ComC knock-out mutant with the wildtype ComC from Var2**. ComC_Var2_ lacks the finger-like domain (see Fig. 4 of the main manuscript), and it was not able to functionally complement the knock-out of ComC_Var1-1._ Please note, we restricted this additional analysis to the two phenotypes that show the strongest effect upon comC deletion, natural competence and piliation. We have updated Figure 5 to show the results of these experiments.

***Figure 5. Functional characterization of ComC in A. baumannii AYE-T.*** *(A) Representative electron micrograph of wild-type A. baumannii AYE-T cells (top) and of the ΔcomC mutant (bottom). No piliation defect is seen for the mutant. A phenotyping of wild-type A. baumannii AYE-T, of the ΔcomC mutant, and of the mutant complemented with the indicated constructs are shown in the following panels. (B)* *Adhesion to HUVEC cells. Bar height and error bar indicate the mean number and standard deviation of colony forming units (CFU) per mL (n=4; values are given as S2 Table). ‘*’ indicates a significant difference (one tailed t test: p<0.05). Complementation with the truncated comC mutants did not significantly increase adhesion rates. (C) Twitching motility. Cells were stained with 1% [w/v] crystal violet. (D) Natural transformation rates of the indicated A. baumannii cells was assessed using genomic DNA of rifampicin resistant A. baumannii ATCC 19606 ^T^. n.d. - not detected.*

In particular, the 3^rd^ experiment, which we have now added, strongly indicates that ComC_Var1-1_ and ComC_Var2_ have functionally diverged, which is the main message of our manuscript. The finger-like domain is the most obvious candidate that could be responsible for this divergence, but we cannot rule out that other differences exist that drive this divergence. We have therefore softened our statements in this direction. We write now in the corresponding part of the Results section:

*The partial restoration of natural competence is an independent albeit indirect indication that the truncated ComC mutants do not show a strong piliation defect. To provide further evidence that the observed phenotypes are also not the effect of more subtle pilus instabilities caused by a truncated ComC, we complemented Ab AYE-T ΔcomC with the wild-type ComC_Var2_ from Ab 17-VT4715T-2. We investigated only the effect of the complementation on natural competence and on twitching, as these phenotypes showed the strongest effect upon comC deletion. Notably, ComC_Var2_ could neither restore natural competence nor twitching (Fig. 5C-D).*

### MI2.1 - Based on figures 1 and 3, it appears that pilV has similar levels of variation and may be covariant with comC and fimU…

*while only variation in the later two is discussed. Do the pilV genes in Var2, Var1-1 and Var1-2 subsets differ?*

Thank you for spotting this, **indeed PilV displays the same pattern of strain-specific differences as ComC and FimU. In brief, PilV exists in two variants**. In Var1, we could annotate an N-terminal methylation motif (PF07963), whereas this motif was not annotated in Var2. This integrates perfectly with our previous observations, because *pilV* is located together with *comC* and *fimU* in the same gene cluster. It is for this reason, that the main message of our manuscript does not change, because all three genes reside in the same evolutionary cassette. Please note that we found on the level of feature architectures an indication that PilV and FimU differ between the Var1-1 and Var1-2 subsets.

In the main text, we have updated the results section, where we write now:

*More compelling are the findings for PilV, FimU and the pilus tip adhesin ComC. These three proteins exist in two main feature architecture variants in the genus (Fig 1D and S6 Fig). PilV_Var1_ differs from PilV_Var2_ by the annotation of a N-terminal methylation motif (PF07963) (…)*

Moreover, we have now also updated Figs. 1D, 2 and S6 Figure and S7 Figure, and we have added a new S12 Figure that shows the comparison of the modelled 3D structures o PilV_Var1_ and PilV_Var2_.

*Figure 1. Characterization of Acinetobacter type IVa pilus components.*

***Figure 2. Maximum likelihood phylogeny of ComC.*** *Red branches indicate A. baumannii isolates and black branches isolates from all other species. The inner ring (ring 1) identifies the taxonomic group the isolates in the tree are assigned to. The variant assignments of PilV, FimU and ComC across the investigated taxa are provided in rings 2 to 4, respectively. The three evolutionary cassettes present in the ACB clade (see main text) are represented in the outermost ring 5 (see main text for further details). A high-resolution version of this tree is given in S7 Figure. ACB – Acinetobacter calcoaceticus-baumannii complex.*

*S6 Figure. Domain architecture changes of ComC, FimU and PilV within the genus Acinetobacter. (A) The main domain architecture (DA) variants of ComC, FimU, and PilV respectively, in the genus Acinetobacter. (B) Feature architecture similarity (FAS) scores between each of the three Ab ATCC 19606 proteins shown in (A) and their orthologs. The protein of Ab ATCC 19606 is used as reference. FAS scores range from a maximum of 1 (identical architectures) to 0 (no shared domains) [9]. FAS score distributions are given separately for isolates inside (red) and outside (blue) of the ACB complex. The asterisk marks the FAS score mean. The average number of instances per protein (IPP) for the individual domains is represented by the bar plot. Pfam domains: VWA_2 - PF13519; Neisseria_Pil_C - PF05567; N_Methyl - PF07963; GspH - PF12019.*

*S7 Figure. Maximum likelihood phylogeny of ComC.* *The evolutionary relationships of the ComC orthologs are reflected in the left cladogram. A. baumannii isolates are indicated by red branches, isolates of all other species are indicated by black branches. The tree including branch lengths is provided to the right of the figure.* *Annotations show from left to right for each Acinetobacter isolate the taxonomic group assignment, the international clonetype, and the strain type according to the Pasteur scheme. Sequence types according to the Pasteur scheme are specified only when more than 10 isolates share the same strain types. Otherwise, they are summarized under ‘other’. The next annotation columns provide for each bacterial isolate the protein variants of ComC, FimU and PilV, and the cassette type. A high-resolution version of this tree is available via figshare: https://figshare.com/articles/figure/_/21967694*

*S12 Figure. Comparison of two PilV variants. (A) Comparison of the modelled 3D structures of PilV_Var1_ (A. baumannii ATCC 19606T) and PilV_Var2_ (A. baumannii 1297). The structures are colored according to their mutual structural similarity from high (blue) to low (red). (B) Pairwise sequence alignment of the two PilV variants. Residue numbering refers to PilV in Ab ATCC 19606^T^. The non-detection of the N-terminal methylation motif (PF07963) in PilV_Var2_ is due to the substitutions at positions 14 (V->A) and 17 (L->M) in the alignment.*

### MI2.2 It is no fault of the authors because the naming of T4P genes and gene-products in the literature is a mess.

*It may be easier for some readers to follow if the gene names of the Pseudomonas homologues (pilA rather than comP, pilY1 rather than comC) were used, simply because that system has been better studied historically. The authors do include both names in some parts of the text, but some genes (ex. comE and comF are included in Figure 1 but not discussed) A simple table of equivalency could be added to Figure 1 to help readers more familiar with the Pseudomonas (rather than the A. baylyi) T4P nomenclature.*

Thank you very much for this very helpful suggestion. We have now updated the table with the gene names of the corresponding proteins from *P. aeruginosa* PAO1. We considered three kinds of evidence to link an *A. baumannii* ATCC 19606 gene with its counterpart in *P. aeruginosa:*

1. Inference of orthology
2. Assignment based on literature evidence
3. Conservation of gene order

For each of the 20 *Ab* ATCC 19606 proteins, we established a link to a protein in *P. aeruginosa* PAO1, if any of the three criteria was fulfilled. The modified Table 1 is shown below.

**Table 1.** Genes involved into T4aP formation in *A. baylyi* ADP1 and in *A. baumannii* ATCC 19606 ^T^.

| Gene name^§^ | A. baylyi  ADP1 | A. baumannii  ATCC 19606 ^T^ | Locus | Annotation |
| --- | --- | --- | --- | --- |
| pilU^&^ | WP_004922051.1 | WP_000347036.1 | 124856-125974 | PilT/PilU family type 4a pilus ATPase |
| pilT^&^ | WP_011182164.1 | WP_000220756.1 | 126002-127069 | type IV pilus twitching motility protein (pilus ATPase) |
| pilF^&^ | WP_011182074.1 | WP_000537691.1 | 636841-637608 | type IV pilus biogenesis/stability protein PilW |
| pilB^&^ | WP_004920473.1 | WP_001274985.1 | 831958-833670 | type IV-A pilus assembly ATPase PilB |
| pilC* | WP_004920476.1 | WP_000279216.1 | 833700-834926 | type II secretion system F family protein |
| pilD^&^ | WP_004920478.1 | WP_001152283.1 | 834926-835786 | prepilin peptidase |
| comM/pilM^&^ | WP_004923715.1 | WP_000537766.1 | 1511699-1512721 | pilus assembly protein PilM |
| comN/pilN^&^ | WP_004923716.1 | WP_000201227.1 | 1512721-1513362 | PilN domain-containing protein |
| comO/pilO^&^ | WP_004923719.1 | WP_000076101.1 | 1513359-514099 | type 4a pilus biogenesis protein |
| comL/pilP^&^ | WP_004923726.1 | WP_000695065.1 | 1514110-1514637 | pilus assembly protein PilP |
| comQ/pilQ^&^ | WP_004923730.1 | WP_001017037.1 | 1514700-1516865 | type IV pilus secretin family protein |
| comP/pilA^&^ | WP_004923779.1 | WP_000993713.1 | 1529663-1530091 | pilin |
| fimU^&^ | WP_004923829.1 | WP_001214059.1 | 1539389-1539862 | prepilin-type N-terminal cleavage/methylation domain-containing protein |
| pilV^&^ | WP_004923832.1 | WP_002194578.1 | 1539856-1540416 | type IV pilus modification protein |
| comB/pilW^#^ | WP_004923834.1 | WP_000079195.1 | 1540417-1541418 | PilW family protein |
| pilX^$^ | WP_004923837.1 | WP_086221418.1 | 1541475-1542233 | pilus assembly protein |
| comC/pilY1^$^ | WP_004923840.1 | WP_085940514.1 | 1542341-1546099 | VWA domain-containing protein |
| comE/pilE^&#^ | WP_004923843.1 | WP_001046417.1 | 1546112-1546594 | prepilin-type N-terminal cleavage/methylation domain-containing protein |
| comF/pilE^#^ | WP_004923844.1 | WP_000788344.1 | 1546591-1547016 | prepilin-type N-terminal cleavage/methylation domain-containing protein |
| fimT | WP_004922618.1 | WP_000477156.1 | 1756167-1756682 | type II transport protein GspH |

§ gene name in *P. aeruginosa* is given after the ‚/‘ if it differs from the gene name in *A. baylyi*
* Pseudogene in *P. aeruginosa*

& inferred by orthology

$ connection established by gene order conservation
\# *P. aeruginosa* *pilE* is co-orthologous to *A. baylyi* *comE* and *comF*

### MI2.3 In Figure 1C, I am concerned that the figure overstates the differences between some of these genes.

*As the authors correctly point out, the absence of a pfam domain could simply be lack of recognition. I do not believe, for instance, there are any Acinetobacter fimT or pilX genes which lack the N-terminal PilD recognition site or TM helix which make up the N-Methyl domain. Similarly, I would imagine that all Acinetobacter fimU genes are gspH homologues based on the commonly proposed models for the T4Pa tip complex.*

Thank you very much for this comment, however we would like to bring to the reviewer’s attention that **Fig. 1C summarizes the outcome of a bioinformatics analysis**. **We simply display the Pfam domain architectures of the proteins as they are represented in *A. baumannii* ATCC 19606 alongside with notable deviations observed in other taxa (Fig. 1D).** At this point in the analysis, we do not make any statements concerning the possible relevance of these differences. Instead, we use these results for shortlisting candidate proteins that we then investigate in greater detail.

The reviewer explicitly mentions FimT. We had already shown in S4 Figure that *Ab* ATCC 19606 features a substitution at an otherwise highly conserved position (see our answer to M3.4.2). The resulting sequence is no longer similar above threshold to the N-methyl pHMM from Pfam, and hence this motif is no longer annotated. We now have added an analysis, which reveals that not only the N-terminal methylation motif is no longer detected, but that the substitution modifies the pre-pilin peptidase cleavage site. This might be the reason why *A*b ATCC 19606 does not twitch. These new results are now summarized in a new S5 Figure, and to avoid redundancy in this answer letter, we would like to point to our answer to MI1.5 above, where we also show the new S5 Figure.

### MI2.4 - In Figure 4 (panels A and B), it is difficult to follow what is happening with the superimpositions at this figure resolution

*I am concerned that some of the differences are artifacts of attempting to align the whole multi-domain structure. Structure prediction (including AlphaFold) is much better at predicting domain structure than aligning multiple domains and for some (ex. ComC from A. baumannii NIPH 1734 in Figure 4A) the domains may simply be rotated (arbitrarily) the wrong way in the model.*

We agree with the reviewer that such structure alignments need to be taken with a grain of salt. However, we think that we were very careful in avoiding issues arising from difficulties with aligning structures from multi-domain proteins. In brief, we

1. Let AlphaFold2 model the structures of each protein independently.
2. Visualize the models with VMD [10].
3. The new **S11 Figure shows that the confidence of AlphaFold2 in the structural models is very high overall, and this is particularly true for the finger-like protrusions**.
4. Use STAMP to align the full models for the various ComC against ComC*_Ab_* _ATCC 19606_. This information in shown in Fig. 4A.
5. Use the MultiSeq extension from VMD [11] to assess the structural similarity between the aligned structures shown in Fig. 4A. We then color-code the structural model of the individual ComC proteins to reflect the extent of structural similarity to ComC*_Ab_* _ATCC 19606_. This information is shown in Panel 1 of Fig. 4B where red parts in the structure represent regions where the two compared structures do not align well.
6. **Because (4) and (5) might be confused by the multi-domain nature of ComC, just as the reviewer pointed out, we next aligned the N-terminal and the C-terminal part of ComC separately [12]**. We manually removed either the N- or the C-terminal parts of the two compared ComC. We then repeated steps (4) and (5) with the remaining structures. Please note, this latter step should avoid that the multi-domain nature of ComC interfers with the structural alignment. The results are shown in Panels 2 and 3 of Fig. 4B. We would like to **emphasize that the structural similarity between ComC*_Ab_* _ATCC 19606_ and ComC*_Ab_* _NIPH 1734_ increases when we look at the N- and C-terminal domains separately** (overall: Id=13.6%; N-terminal: Id=16.3%; C-terminal: Id=22.6%).

***Figure 4. Variation of ComC in A. baumannii.*** *(A) Alignments of the modelled 3D structures for Ab ATCC 19606 ^T^ ComC (blue) and the ComC of other bacterial isolates (red). The per-residue confidence for each modelled structure is given as the pLDDT value below the structures. Values >90 indicate a high accuracy and values between 70 and 90 a good accuracy of the prediction [13]. (B) Extent of structural conservation between ComC proteins shown in (A) and the reference protein in Ab ATCC 19606 ^T^. The structure of the target protein is colored with a gradient from blue (high conservation) to red (low conservation). Q_H_: pair-wise structural conservation score ranging from 1 (structurally identical) to 0 (no similarity). ID: percent of sequence identity in the structural alignment. (C) Multiple sequence alignment of the N-terminal part of representatives for the three ComC variants in A. baumannii. The alignment covers the amino acids 24 – 233 of Ab ATCC 19606 ^T^ ComC. The sequences forming the finger-like protrusions in ComC_Var1-1_ and ComC_Var1-2_ are shaded in brown. The sequences for the three variants represent the corresponding species shown in (A). (D) Structural similarity between the finger-like protrusions of ComC_Var1-1_ and ComC_Var1-2_. The color gradient from blue to red indicates decreasing similarity. The tyrosine-rich motif is highlighted in green. (E) Pair-wise sequence alignment between the sequences forming the finger-like protrusion in ComC_Var1-1_ and ComCVar_1-2_. Conserved residues are indicated in dark blue; the tyrosine-rich motif is shown in green.*

To conclude this part, **we are confident that the different orientation in 3D space of the finger-like protrusion in ComC_Ab ATCC 19606_ and in ComC_Ab NIPH 1734_ is not an artifact of the structural alignment**. In combination with the different positions of the sub-sequences forming the fingers (see Fig. 4 and S14 Figure) and the different placement of the corresponding proteins in the ComC phylogeny (see Fig. 2), this is evidence that we are looking at two structurally and evolutionary distinct variants of ComC. To what extent they differ in function remains to be determined, and we make this now clear in the discussion. **We write now**:

*The observation that wild-type ComC_Var2_ could not complement the deletion of ComC_Var1-1_ further supports this view. Still, our evidence is only preliminary, and further analyses will be necessary to prove the involvement of this finger in ComC_Var1-1_ function. It will then also be interesting to see whether ComC_Var1-2_ differs in function from ComC_Var1-1_, and whether the Tyr-rich motif, that is present in the fingers of both variants has a functional role.*

## Reviewer 3

*Reviewer #3: This manuscript by Iruegas and colleagues deals with comparative genomics of T4aP systems in Acinetobacter genomes at the protein-domain level, in order to find determinants of pathogenicity in A. baumannii, whenever more classical comparative genomics involving gene repertoires could not find relevant traces. The authors claim to find some specificities in terms of domain compositions for pathogenic strains, and make some experimental testing on the role of these domains in abilities of Acinetobacter to adhere to human cells, to perform twitching motility or competence. The idea is appealing and interesting, as it is true that the information at the domain-level is very often overlooked in comparative genomics, even though it may hold crucial cues to understand bacterial adaptation. However, the manuscript is difficult to read and follow, as the bioinformatic methods in particular, and even the global approach is hard to grasp. In particular, it is unclear how protein domains were annotated (see my comments) and how a “domain loss” (or addition) was inferred, whenever it is crucial for the analysis that look into the evolution of the presence/absence pattern of protein domains. In the end, I am still wondering what really makes the difference between the sequences of the protein-domain variants of FimU and ComC. And can the outcome of a PFAM domain annotation suffice to infer the presence or absence of a functional domain? Considering the potential thresholds effects in such an analysis, I would assume that it should be completed with in-depth multiple sequence alignment analyses.*

*It is also a bit difficult to understand how the recombination and protein domain analyses results articulate. I finally also wonder how reliable are the structural modelling experiments, and whether the experimental designs proposed to test the functional role of ComC domains highlighted by bioinformatic analyses, are sufficient to support the conclusions, in particular that the authors “provide experimental evidence that this finger conveys virulence-related functions in A. baumannii” (from abstract, see also my comments).*

*For these reasons, I unfortunately cannot be supportive of the publication of this article. I think the work and approach is valuable and of interest, but that there are missing pieces to make it a convincing case, and an effort to be made on how the results and methods are presented. A synthetic figure on the evolutionary proposed for example could be valuable. Please find more details in my comments below, I hope they will be considered as constructive.*

### General remarks of the authors

We are grateful for the critical assessment of our study, and for pointing out where we can improve in explaining the rationale, the approaches, and in highlighting strengths of our study.

- We have the impression that the most prominent issue lies in the distinction between a ‘**non-detection**’ of a sequence feature, e.g., a ‘Pfam domain’, and the **absence** of a structural domain together with its function. In particular, the **dual use of the term ‘domain’ requires that ‘domain gain’ and ‘domain loss’ need to be interpreted differently depending on which kind of ‘domain’ they refer to**. We tried to make this clear in the original version of our manuscript **by always explicitly referring to a ‘Pfam domain’, when ‘absence’ should be equated with ‘non-detection’**. However, guided by the reviewer’s comment, we have become now more precise in this matter.
- The second major issue we see, is that the **hierarchy of our analyses is not clear**.

We use this opportunity to quickly recapitulate the workflow, which is based on a new bioinformatics approach that allows the comparison of protein feature architectures between orthologs [14]. We first **introduce a new term ‘feature’**, which is an amino acid (sub-)sequence whose characteristics allows a dedicated annotation software to assign a specific label to them, i.e., the name of the feature type. Here, we consider for example Pfam- and SMART domains, low complexity regions, and transmembrane regions as ‘features’ [14]. A feature architecture is then the arrangement of features from N- to C-terminus of the protein. We have updated the introduction with a definition of the term ‘feature’. **We write now:**

*Introduction*

*Therefore, it is still unclear to what extent T4aP* *di**ffer between members of this genus and what consequences this may have for the interaction of A. baumannii with the human host.*

*Here, we increase the resolution of the comparative analysis of T4aP components to the level of individual protein features, such as the presence of Pfam- or SMART domains, of transmembrane domains and of low complexity regions [14]. For each protein, we integrate the annotated features from N- to C-terminus into a feature architecture and compare these between orthologs of the T4aP components. We then exploit that differences in the feature architectures of two orthologs serve as a proxy of their functional divergence [14].*

Additionally, we now write in the Results section:

*The phylogenetic profiles provide no evidence for a lineage-specific modification of T4aP within Acinetobacter that is driven by the gain or loss of individual genes. We therefore increased the resolution by comparing the feature architectures of the Ab ATCC 19606 ^T^ T4aP components to those of their orthologs (Figs 1B-D).*

**We next explain the individual levels in our bioinformatics analysis workflow. Please note, the sensitivity of the analysis increases with each level,** i.e., we become better in identifying differences between orthologous proteins across bacterial isolates. **However, the specificity decreases**, i.e., an increasing fraction of observed changes may have no functional consequence or just represent noise. **This requires additional analyses to validate the (functional) relevance of the observed changes**. Because these additional analyses are time- and cost-intense, they should be reserved to promising candidates.

- **Level 1** - Analysis of the presence/absence pattern of orthologs across bacterial isolates. We see no difference between T4aP components across *Acinetobacter isolates*. This information is shown in Fig. 1B
- **Level 2** - Comparison of feature architectures between Ab ATCC 19606 proteins and their orthologs. We find 5 candidates with marked changes in their feature architecture). This information is shown in the color coding of Fig. 1B and in Figs 1C-D
- **Level 3** - Integration of the phylogenetic profiles with the information about feature architecture changes (we reduce the number of candidates to 3: ComC, FimU and PilV). This information is shown in Figs. 1B-D
- **Level 4** – Maximum likelihood tree reconstruction integrated with the recombination analysis. This revealed that ComC, FimU, and PilV are part of an evolutionary cassette represented by 3 distinct evolutionary lineages in A. baumannii. This information is shown in Figs. 2 and 3
- **Level 5a** Comparison of the 3D structure models. In the case of ComC, this reveals that the overall structure of the variants is similar, but that the most prominent difference is the presence of a finger-like structure in Var1, which is not seen in Var2. This information is shown in Fig. 4
- **Level 5b** - Multiple sequence alignment to see how changes in the feature architecture, differences in the modelled 3D structure integrate with differences on the amino acid sequence level. This reveals, for example, that the sequences supposedly forming the finger-like structure in ComCVar1-1 and ComCVar1-2 are most likely not homologous. This information is shown in Fig.4 for ComC
- **Level 6** – VAST search in PDB for sequences showing a local structural similarity to the individual ComC variants. This search was limited to ComC, and revealed in all cases structural similarities in the N-terminal region to proteins with a vWFa domain. See M3.4.3 for further details here.

ComC came out as the most promising candidate of our bioinformatics comparison of 20 T4aP components across more than 850 bacterial isolates. Because bioinformatics analyses remain predictive, we then provide a first **experimental characterization** of ComC where we show that

- A knock-out of *comC* affects adherence of A. baumannii AYE to human cell, abolishes twitching and natural competence
- A complementation with wild-type Ab AYE ComC rescues the three phenotypes
- A complementation with mutant ComC lacking either the entire von-Willebrand-Factor A domain, or lacking the sequence forming the finger-like protrusion cannot rescue adhesion or twitching, but it leads to a partial restoration of natural competence.
- **We now also show that a complementation with wild-type ComC Var2 does not rescue the twitching and natural competence phenotype**.

**To conclude this aspect,** we hope that this summary has sets the stage for the following point-by-point answers to the individual issues raised by the reviewer.

### M3.1 - Line 131. “Orthologs to all”... Please start the paragraph saying a word on the methodology for "ortholog search", otherwise the entire paragraph is hard to follow.

*As this is so central to this study’s methodology and results, I believe it should be explained what it consists in, and that providing a link to a Github repository is not sufficient in Methods (Line 396). Are you sure they are orthologs and follow the same gene history? How was it inferred? If not inferred, then maybe could the less precise "homologs" term be used?*

Thank you very much for this comment. **To provide the facts first**: We use fDOG for identifying orthologs to the 20 proteins of interest across more than 850 bacterial isolates.

- fDOG is a **successor of the targeted ortholog search tool HaMStR [15]**. HaMStR has been cited more than 200 times (Web of Science) since its publication often by studies that are interested in the compilation of phylogenomic data sets, i.e., by studies where both scalability but also the accuracy of the ortholog search are crucial parameters. **fDOG and HaMStR share the core functionalities of the ortholog search**, with fDOG having of course the more modern implementation. As one key innovation, fDOG is the only ortholog search tool that can assess the similarity of two orthologs on the level of their feature architectures [14].
- The manuscript that describes fDOG is currently in preparation, but we are happy to already now share the benchmark with the reviewer where **we compare fDOG with two state-of-the-art ortholog search tools, InParanoid [16] and OMA [17] using the Quest for Orthologs benchmark server [18] (Fig. AR3).** In brief, we use the three tools to identify orthologs for all human proteins in 77 Quest for Orthologs reference proteomes [19]. **fDOG shows a comparable accuracy with these tools, but it can search for orthologs in several thousands of gene sets (e.g., [4, 20]).**

*Fig. AR3. Benchmark of fDOG in comparison to OMA and InParanoid. The benchmark was performed using pair-wise orthology assignments between human proteins in the H. sapiens QFO reference proteome and each of the remaining 77 species in the set of QFO reference proteomes. We show the results for three benchmark challenges: (i) The extent of semantic similarity for EC annotated orthologs (panel 1), (ii) the extent of semantic similarity of the GO annotation of the annotated orthologs (panel 2), and (iii) the Robinson-Foulds distance between the gene tree reconstructed from the orthologs and the corresponding species tree (panel 3). The X-axis in the plots below provide an indication about the inclusiveness of the tool, whereas the respective Y-axes inform about the specificity. For panels 1 and 2, the highest achievable score is 1, when the functional annotations of the ortholog pairs are always identical. For panel 3, the optimal score is 0, when all gene trees are congruent to the species tree. For each ortholog pair, fDOG assigns a feature architecture similarity (FAS) score between 0 (no shared features) and 1 (identifical architectures) [14]. fas75_f and fas75_r are subsets of the ortholog pairs where the FAS score is at least 0.75.*

- We provide with the github repository not only access to the source code of fDOG but also a thorough documentation of its functionality, such that readers can reproduce our finding

To conclude this point, fDOG is a highly accurate state-of-the-art tool for a target ortholog search in large and phylogenetically diverse taxon collections. The key innovation of fDOG compared to its predecessor HaMStR is the automatic integration of domain architecture similarity scoring between the seed protein and its orthologs using an algorithm that has just been published in Bioinformatics [14]. We updated the corresponding paragraph in the methods section where we now write:

*Targeted ortholog searches were performed with fDOG v0.0.8 (*[*www.github.com/BIONF/fDOG*](http://www.github.com/BIONF/fDOG)*), an extended version of HaMStR [15] using A. baumannii ATCC 19606 ^T^ as the reference taxon. Feature architectures similarities between orthologs were computed with the software package FAS v1.3.2 (*[*https://github.com/BIONF/FAS*](https://github.com/BIONF/FAS)*) [14] that is integrated into the fDOG package.*

In the first paragraph of the Results section, we start the corresponding paragraph now with:

*We first performed a targeted ortholog search for the individual Ab ATCC 19606 ^T^ T4aP components across 855 Acinetobacter isolates that cover the diversity of the genus complemented with 29 outgroup species. The resulting presence/absence patterns of orthologs are summarized in the phylogenetic profiles shown in Fig 1B.*

### M3.2 - Line 152-153: rather than “chance effects” that sounds a bit odd, couldn’t the authors rather involve variations in sequences…

*to explain the annotation or not of this domain with PFAM profiles? Depending on their specificity and sensitivity?*

When writing ‘chance effects’, we exactly had changes in the amino acid sequence in mind. In the mentioned cases, the similarity between the amino acid sequences and the pHMM was already only slightly above threshold. In essence, individual substitutions may suffice to slightly reduce the similarity to the pHMM below the annotation threshold. The cases where the feature is then not annotated are then randomly distributed across the investigated taxa. We have rephrased this part now and write:

*The feature architectures of many PilX orthologs differ from that of the protein in Ab ATCC 19606^T^ by the absence of a PilX N-term Pfam domain (PF14341; see Fig 1C-D). Upon closer inspection we found that in those proteins, where PF14341 could be annotated, E-values often only barely meet the detection threshold (S3 Fig). Thus, the sequence variation of A. baumannii PilX N-termini appears not sufficiently captured in the Pfam pHMM. This explains why this feature is often missed.*

### M3.3 - On line 390 of the Methods, it would be very important to indicate what parameters were used for HMMER?...

*Was the cu-ga option used? Otherwise, which thresholds? And which HMMER version?*

We have added this information to the Methods section. In brief, we used the HMMER package version 3.3.2. *hmmscan* was run with the options -E 0.01 --domE 0.1 –noali. We did not use the option --cut_ga. We acknowledge that tweaking these parameters for individual proteins and features may result in slightly altered feature architectures. However, since the architecture-based pre-screen of the 20 pilus components serves only as an entry into our analysis, **we trust that this effect is conservative when it comes to the main conclusions of our work**. In the methods section, we write now:

*Pfam and SMART domains were annotated with hmmscan from the HMMER package v.3.3.2 [21] with the options -E 0.001 --domE 0.1 --noali using Pfam release 32.0 [22] and SMART release 9 [23]*;

### M3.4 - We touch here the heart of my main criticism:

#### M3.4.1 - I do not understand how domains were annotated in details…

*and how/when a loss of domain was inferred, and how to distinguish it from cases where sequence divergence solely could explain the results.*

We highly appreciate the precision of the reviewer, and we agree that there was room to improve the precision in our description. We see the main reason for confusion in **the ambiguous use of the word ‘domain’, in particular in the context of the von Willebrand Factor A domain. We infer the presence of this domain in the N-terminus of ComC either based on a sequence similarity above threshold to the VWA_2 Pfam pHMM. Alternatively, we use a local structural similarity to proteins carrying a von Willebrand Factor A domain (vWFa) as evidence for the presence of this domain (see S15 Figure).** Please see also our answer to M3.4.3.

We now give a general idea of how we annotate ‘Pfam domains’ in this manuscript. Please note, that Pfam domains are now subsumed under more general term ‘feature’ (see General remarks)

- We use ‘Pfam domain’ in the **context of a sequence family that is modelled by a profile Hidden Markov Model** **by Pfam**. To clarify the issue of how we annotate ‘Pfam domains’: For a protein ***P*** represented by its amino acid sequence, and a pHMM named ***Y*** provided by Pfam, **we annotate *P* with *Y* if *hmmscan* reports a significant local similarity between the amino acid sequence of *P* and the pHMM representing *Y* that passes the Bit score and E-value cutoffs**. In this case, we sometimes wrote a bit loosely “*Pfam domain* ***Y*** *is present in* ***P***”. If no significant similarity is detected, ***P*** is not annotated with ***Y***, and we stated that “***Y*** *is absent from* ***P****”*. If orthologs of ***P*** are annotated with ***Y*** but not ***P*** itself, then we considered ***Y*** as either ‘lost’ in ***P*** or ‘gained’ by the orthologs of ***P***.

**We now updated the manuscript and made sure to use only the term ‘annotation’ in the context of Pfam domains. Moreover, we always prepend ‘Pfam’ when we refer to a feature that has been annotated with the help of a pHMM provided by the Pfam database.**

- We **also refer to structural units in a protein as ‘domains’**, e.g., the N- and C-terminal globular domains identified in the structural model of ComC that was produced with AlphaFold2, or the vWFa domain that has been described in [24]. Concerning structural domains, we identify them based on 3D models of the protein structures or by using similarity searches in PDB with the help of VAST [25]. In comparative analyses between two proteins, we consider a domain as ‘absent’ or ‘lost’ if the corresponding structural domain is found in only one of the two proteins. One prominent example is the finger-like protrusion, which we find in ComC_Var1_.

To conclude this point, we have updated the manuscript such that **we now clearly differentiate between cases where we did not annotate/identify a certain feature represented by a Pfam pHMM and those cases where we infer the gain/loss of a domain and of its function.** Moreover, we always explicitly refer to a ‘**Pfam domain**’, when we write about the (non-)detection of a sequence feature that is similar below/above threshold to a pHMM provided by Pfam. We hope that this increases the precision of the manuscript.

#### M3.4.2 - For example on lines 156-158 the authors then claim themselves “The domain loss in the A. baumannii type strain is caused by a substitution…

*that results in a tyrosine at a position that is typically occupied by a leucine or an isoleucine in PF07963”. Rather than invoking here and elsewhere “domain losses”, could the authors rather involve threshold effects in the annotation? That the domain for example does not pass the threshold?*

We hope that our answer to the previous point addresses also this part. Throughout the manuscript, **we now very carefully differentiate between the likely non-detection of a feature due to threshold effects, and the absence/loss of a feature and of its function**. We demonstrate this in this answer letter using PilX (threshold effect) and FimT (loss) as examples.

PilX is an example for a threshold effect affecting the feature annotation of PilX, **we write now**:

*The feature architectures of many PilX orthologs differ from that of the protein in Ab ATCC 19606^T^ by the absence of a PilX N-term Pfam domain (PF14341; see Fig 1C-D). Upon closer inspection we found that in those proteins, where PF14341 could be annotated, E-values often only barely meet the detection threshold (S3 Fig). Thus, the sequence variation of A. baumannii PilX N-termini appears not sufficiently captured in the Pfam pHMM. This explains why this feature is often missed.*

FimT, in turn, is an example for a very likely feature loss. To remind the reviewer, we could not detect an N-terminal methylation motif (*PF07963)* in individual FimT orthologs. Our analysis comprised two steps. In Step 1, we showed that an I->T substitution in FimT of *Ab* ATCC 19606 changes a conserved amino acid position within a prepilin peptidase recognition motif. According to the pHMM for PF07963, **threonine has an emission probability of 0 at this position**. This explains why the Pfam domain is not annotated in these proteins. **This information is summarized in S4 Figure** (see below). The reason why we think that the findings of Step 1 is not a result of an overfitted pHMM, as it appears the case for PilX, is the following: In step 2 of our FimT analysis, we show that an I->T change is the sole difference between FimT of two non-twitching A. baumannii strains compared to twitching strains. Please see our answer to MI1.5 for further details on this matter.

*S4 Figure. Characterization of the pilin FimT.* *(A) Pair-wise feature architecture similarities between Ab ATCC 19606^T^ FimT and its 864 orthologs across the analyzed taxa [9]. The upper block indicates the feature architecture similarity (FAS) scores penalizing the non-detection of an Ab ATCC 19606 feature in the ortholog (FAS_F). The consistent coloring in blue indicates that the overall domain architecture similarity is high. The lower block penalizes the absence of a feature that is seen in the ortholog but that is absent in Ab ATCC 19606^T^ FimT (FAS_B). Most orthologs are colored in pink, which indicates that they possess a domain that is not annotated in Ab ATCC 19606 ^T^ FimT. (B) Pairwise feature architecture comparison between FimT in Ab ATCC 19606 ^T^ (WP_000477156.1) and its ortholog in Ab AYE (WP_000477147.1). This reveals that Ab ATCC 19606 ^T^ FimT could not be annotated with the N-terminal methylation motif provided by Pfam (PF07963; purple). (C) Pairwise sequence alignment between FimT in Ab ATCC 19606 ^T^ and in Ab AYE. Conserved residues are highlighted in dark blue; substitutions are shown in light blue. FimT of Ab ATCC 19606 ^T^ features a threonine at position 9 in the alignment. (D) The HMM weblogo of the N_methyl domain reveals that position 9 is typically occupied by either leucine or isoleucine. (E) Emission probabilities for the 20 amino acids at position 9 in the pHMM representing the Pfam N_methyl motif (PF07963). The emission probability for threonine is 0 at this position, which explains why no N_methyl domain was annotated in FimT of Ab ATCC 19606 ^T^. ACB – Acinetobacter calcoaceticus-baumannii complex; γ – γ-proteobacteria; β – β-proteobacteria.*

#### M3.4.3 - Again, what is unclear to me here and later on, is whether the domain is really “lost” or gained…

*(and what would mean lost here, it is not an entire domain deletion apparently from figure 1C-D?) or whether the sequence is just divergent from the consensual domain as defined in PFAM. In Discussion (line 314-319), the authors themselves observe: “However, general features of a VWA domain are present also in ComC of a-pathogenic Acinetobacter isolates.” And further ask “Why then were these domains not annotated with the VWA_2 Pfam domain?”*

We hope that our explanations up to now already helped clarifying this aspect. Initially, we use the non-detection of a Pfam domain in a sequence as an indicator for a change that might be of functional relevance. **For individual candidates, we then differentiate between two cases:** In **case 1**, **we overlook a Pfam domain** due to threshold effects (see PilX as an example). In **case 2**, **the non-detection indicates changes on the amino acid sequence level that are likely have a functional consequence** (see FimT). In the case of the N-terminal part of ComC, the situation is a bit trickier, because we must consider both cases at the same time. To set the stage, we differentiate between

1. an amino sequence showing a similarity above threshold to the VWA_2 Pfam domain. To describe this case, we refer to the annotation of a VWA_2 domain.
2. an amino acid sequence that can assume a 3D structure that is significantly similar to a von Willebrand Factor A domain as it is described in the literature. To describe this case, we refer to the presence of a vWFa domain

In our study, all investigated ComC share the presence of a vWFa (see S15 Figure), but only a subset is annotated with a Pfam VWA_2 domain. Thus, the **Pfam VWA_2 pHMM does not cover the full space of sequences that can assume the structure of a von Willebrand Factor A domain.** To further back this claim, Siryaporn et al. reported the presence of a VWFa domain in *P. aeruginosa* PilY1 [26], but this sequence shows no similarity above threshold to the Pfam VWA_2 pHMM.

To conclude this part, in the revised manuscript **we now clearly differentiate between the ‘non-detection’ of a domain resulting in the change of the feature architecture, and its absence/loss**. In the Discussion, we write now:

*On the first sight, our findings that exclusively ComC of pathogenic Acinetobacter could be annotated with a Pfam VWA_2 domain suggest that the acquisition of a vWFa domain drives the conversion of T4aP into a virulence factor.*

*However, the situation appears more complex. Higher-resolving analyses indicate that also ComC of a-pathogenic Acinetobacter isolates possess a vWFa domain although the corresponding amino acid sequence is not similar above threshold to the VWA_2 Pfam domain. Evidences include the presence of the characteristic metal ion-dependent adhesion (MIDAS) motif (see S14 Fig; [27]) as well as extended stretches of local structural similarity between the N-terminal region of ComC and eukaryotic vWFa containing proteins (see S15 Fig). What however differentiates the investigated ComC variants are independent insertions into the conserved structural scaffold formed by a vWFa domain (see S14 Fig and S15 Fig). In ComC_Var1_ such an insertion resulted in the formation of a finger-like protrusion (see Fig 4 and S15 Fig). Thus, rather than the presence of a vWFa domain in the PTA it might be the variant of the vWFa domain that determines whether T4aP are involved in virulence related functions, or not.*

#### M3.4.4 - It probably does not change the functional interpretation, this domain diversification might be as well very functionally relevant…

*, but it changes the angle of the overall approach here and the relevance of the underlying bioinformatic analyses.*

We thank the reviewer for sharing her/his thoughts with us, as this helped us to sharpen our argumentation. When it comes to the relevance of the bioinformatics analysis, we hope that we are sufficiently clear in stating that our comparative analyses across more than 855 bacterial isolates serves to identify candidates whose feature architectures **have changed to an extent that it renders a time- and cost-intense downstream candidate validation a promising venture**. A **key technology in this context is a state-of-the art bioinformatics algorithm that allows the scoring of feature architecture similarities with an unprecedented accuracy [14]**.

### M3.5 - On lines 166, it is indicated that “We next investigated the evolutionary histories of ComC and FimU in greater detail.”…

*However, I would have expected to see, aside from (or maybe even before) these phylogenetic analyses, an analysis of the multiple sequence alignment of these proteins first, because from the schemes on Fig 1C, it seems that there was not a clear-cut “domain” loss, as the sequences from both variants in both proteins show consistent length. Do they align well? If not, how could it impact the phylogeny? Do the variants clearly differ in sequence at the position of these domains? There is an alignment displayed in Fig4C, but it comes late in the article, and is limited to three sequences, making it difficult to infer how conserved are the different regions of the MSA.*

From our answers to M3.4, we hope that it became now clearer why feature architectures can differ between orthologs but still the protein lengths are comparable. We would like to take the opportunity to remind the reviewer that **our scan for a functional divergence between bacterial isolates acts on different levels** (see the General remarks above). We consider sequence alignments only at a level in the bioinformatics workflow where their very high sensitivity in detecting differences fits into the scope of the analysis.

The reviewer suggests showing a multiple sequence alignment to provide evidence for a ‘domain loss’. While we are ready to provide such an alignment, we have to say that we do not consider this as helpful for the following reason. At this stage of the analysis (see General remarks), we use lineage-specific changes in feature architectures as an **initial indication that the underlying amino acid sequences of orthologs have changed to an extent that it may impact protein function** (see [14]). A multiple sequence alignment does not provide this information unless the corresponding parts in the sequence have been deleted, which is not necessarily the case (see FimT).

### M3.6 - Fig. 2 represents a Maximum-Likelihood phylogeny, but neither branch lengths nor branch supports appear…

*Was there any attempt to root it? These reasons and questionings leave this tree difficult to use for evolutionary interpretations.*

Thank you very much for this comment, and we would like to explain the reasoning why we decided to not show this information. The **sole purpose of this tree is to show that the evolutionary history of ComC leaves *A. baumannii* paraphyletic**, *i.e.,* there is no split in the tree that groups all *A. baumannii* isolates to the exclusion of the remaining species. Branch lengths are not helpful here, because they make the tree harder to read. **To address the reviewer’s comment, we now provide the tree with branch lengths in an updated S7 Figure (**please see M1.1**)**.

**The tree shown in Figure 2 is outgroup-rooted.** We placed the root on the branch leading to one of the two outgroup clades (highlighted in green in the tree), however this is just **for illustration purposes**. **The position of the root is irrelevant, because the paraphyly of *A. baumannii* can be seen also in an un-rooted tree**.

Bootstrap support values are not shown, because they are not helpful in this context and would additionally clutter the figure. **We trust that the reviewer is concerned about the fact that the data might not have enough power to differentiate between monophyletic and paraphyletic *A. baumannii***. We would like to emphasize that we **applied phylogenetic topology testing** to show that a tree with monophyletic *Acinetobacter* or with monophyletic *A. baumannii* explains the ComC alignment significantly worse than the maximum likelihood tree, and thus can be rejected. We made this clearer in the main text. **We write now**:

*A phylogenetic analysis of the ComC orthologs revealed that the species A. baumannii and the genus Acinetobacter are both paraphyletic regarding this locus (Fig 2 and S7 Figure). To exclude the possibility that the paraphyly is an artefact of insufficient phylogenetic signal in the data, we confirmed that an alternative tree where the Acinetobacter isolates were forced into monophyly explains the data significantly worse (AU test; p=0.002; [28]).*

To still give access to the full data, **we now provide the tree including branch lengths in S7 figure, and in Newick format** including bootstrap values and branch lengths together with the underlying alignment **as supplementary data**.

### M3.7 - A recombination analysis followed, but it is difficult to grasp how its results articulate with that of the domain analyses:

*recombination analyses showed a large tract of recombination spanning several genes (Fig. 3) and yet in Fig S11, it is written in the legend “This indicates that a structural backbone of a VWA domain is modified, in a lineage-specific way, by insertions of different origins.” Could the authors clarify? How many events are invoked for instance?*

The results of the recombination analysis, which are shown in Figure 3, provide evidence that the three focal genes of this study, *comC*, *fimU* and *pilV* form together with the remaining T4aP associated genes in this gene cluster an evolutionary unit, at least in A. baumannii. This unit – we refer to it as an *evolutionary cassette* - exists in different layouts that are defined by the feature architectures of ComC, FimU and PilV. The different layouts are named according to variants of ComC that have been detected using the feature architecture comparison integrated with the ComC phylogeny. We refer to the blue lineage as ‘Var1-1’, to the dark-blue lineage as ‘Var1-2’ and to the yellow lineage as ‘Var2’. The remaining colors identify lineages detected by fastGear that do not comprise *A. baumannii* isolates.

**Figure 3. Patterns of ancestral recombination in the locus surrounding the *comC* gene.**

Unfortunately, **we have no clear idea how to comment on the connection between the recombination analysis shown in Fig. 2 and the information shown in S11 Figure (now S15 Figure)**. In S15 Figure, we used a VAST analysis to investigate the N-terminal parts of ComC orthologs spanning the Acinetobacter diversity but also in representatives of other genera. Specifically, we were searching structural databases for sequences displaying a local structural similarity to the ComC ortholog. This revealed that all ComC orthologs display a local structural similarity to eukaryotic VWA containing proteins. However, it also showed that ComC orthologs differ by the presence of sequences with weak structural similarities to a diverse set of proteins in PDB. From this we conclude that all ComC orthologs share a structural backbone of a vWFa domain which is then modified, in a lineage-specific way, by insertions. Specifying how many events are necessary to explain the diversity of the N-terminal region across more than 800 ComC orthologs in our data set might be possible. However, we hope that the reviewer agrees with us that this result would not contribute significantly to the main message of our study.

### M3.8 - Regarding the recombination analysis, the paragraph should contain a word on which principle was used for recombination detection…

*to guide the authors through the results and drawn conclusions. Also, since the authors used phylogenetic approaches, could phylogenies of the genes inside/outside the cassette be used to demonstrate different evolutionary histories, hence supporting the cassette and recombination scenario? Could the phylogeny on Fig 3 already be it? I don’t see it much discussed in the main text. Also, the components from Fig S7 could have been used to complement Fig 3.*

Thank you very much for pointing this out. The data underlying this analysis is a multiple sequence alignment of the sequences included into this analysis, and fastGear investigates what sequences share sequence variants over what part of the alignment. We now state this more explicitly in the main manuscript where we write now:

*This suggests that recombination may have affected the evolution of this locus. We next used the change in the pattern of shared variants between the bacterial isolates to assess the length of the genomic region that was likely involved in these recombination events [29].*

Concerning the additional evidence, the reviewer has suggested to include into the analysis: **We followed the reviewer’s suggestion and computed a further tree based on the concatenation of the genes flanking the T4aP components in Fig. 3.** This tree features monophyletic *A. baumannii* and, thus, lends further support to the recombination hypothesis. This figure is shown as the new S10 Figure.

***S10 Figure. Maximum likelihood phylogeny of the concatenated alignment of the genes flanking the T4aP components****. The gene tree is based on a multiple amino acid sequence alignment of the genes flanking the T4aP components in Figure 3 of the main manuscript. It comprises the following proteins: guanylate kinase (WP_000015937.1), 4-hydroxy-3-methylbut-2-enyl diphosphate reductase (WP_000407064.1), 30S ribosomal protein S16 (WP_000260334.1), ribosome maturation factor RimM (WP_000189236.1), tRNA (guanosine(37)-N1)-methyltransferase TrmD (WP_000464598.1), and 50S ribosomal protein L19 (WP_000014562.1). NCBI accession numbers are given in parenthesis. Branch labels denote percent bootstrap support.*

We now refer to this analysis in the main text, where we write:

*The analysis revealed that the recombination block spans all seven T4aP components in this region but excludes the flanking genes. This observation integrates well with the finding that the gene tree of the concatenated T4aP components leaves A. baumannii paraphyletic (see Fig. 3), whereas the gene tree based on a concatenation of the five flanking genes supports monophyletic A. baumannii (S10 Figure).*

### M3.9 - Line 216-218: “Therefore, the two fingers are very likely of different evolutionary origins…

*although their structural similarity and the shared presence of the Tyr-rich motif suggest that they originated from the same source” I found this sentence unclear and convoluted. Please rephrase.*

We have rephrased the entire section and we trust that this has made the message better accessible. **We write now:**

*Fig 4 shows that both ComC_Var1-1_ and ComC_Var1-2_ share the presence of a finger-like protrusion harboring a Tyr-rich motif. Note that these fingers are embedded into the vWFa domains of both variants, however they are differently positioned both in the predicted structures (Fig 4A-B) and in the respective amino acid sequences (Fig 4C and S14 Fig A-B). Therefore, the two fingers are very likely of different evolutionary origins although their structural similarity and the shared presence of the Tyr-rich motif suggest that they originated from the same source (Fig. 4D-E).*

### M3.10 - I am not a structural biologist, but what is the confidence of the 3D models obtained with AlphaFold in the precise regions of interest for ComC on Fig 4A?...

*I have seen many times these models with mapped confidence scores. Are the features observed to differ between modelled “variants” reliable? What would happen were diverse sequences from a same “variant” chosen for the modelling? What is the confidence in the positioning of the finger-like protruding domain? Or its presence even? Unfortunately, additional information might be present on Fig S11, but the resolution of the image is too low. Could the “finger-like protrusion” region be somehow highlighted or pointed at where relevant on Fig 4?*

We agree with the reviewer that the part about the differences between the modelled structures of ComC is only then relevant if the confidence in the reconstructed structure is sufficiently high. **Structures produced by AlphaFold assign to each position a value that is indicative of structural stability, referred as pLDDT**. We provide average pLDDT values below each of the modelled structures (Figure 4A). **These values indicate a high to very high confidence in the structure reconstruction**. ComC of *A. baylyi* obtained the lowest pLDDT value (74.8), which still falls into the range of a good backbone prediction [13]. Concerning the reviewer’s comment, we prefer to show the overall per-residue pLDDT value in Fig. 4 of the main manuscript instead of cherry-picking values that fit our message. Moreover, we use color-coded structures to display the extent of structural similarity assessed from a structural alignment. However, **considering the reviewer’s advice, we provide now also the structural models with the mapped per-residue pLDDT values as S11 Figure**. This reveals that in particular the finger-like protrusion together with its positioning in the overall structure is modeled with maximum confidence. We understand that this does not prove the formation of this finger in vivo. However, even in the (very unlikely) worst-case scenario that the structural modeling wrongly predicts the existence of this finger, the results and conclusions of the evolutionary and functional analyses performed in this study remain valid. This is, because they do not depend in any aspect on the structure being actually formed as predicted. Concerning the results of the VAST analysis (now shown in S15 Figure), we apologize for the poor quality, which resulted from the conversion to PDF.

When it comes to the within-variant variation of the modelled structure, we exemplarily looked at ComC of *Ab* AYE and *Ab* ACICU and recovered the same structural layout as for Ab ATCC 19606.

*S11 Figure. Structural models of ComC of the indicated bacterial isolates. The color coding in the structures represents the per-residue confidence scores (pLDDT) provided by AlphaFold2. All structures are arranged such that the N-terminal domain is oriented to the left and the C-terminal domain to the right.*

### M3.11 - The proposed mutant comC to test experimentally are based on mutant proteins that seem somehow drastic versions of it….

*Despite the fact that 3D models predict similar folding, again what is the confidence for it?*

The confidence in the predicted structure for the mutant lacking the finger-like protrusion is high (pLDDT= 90.06), and we provide this information now in the caption of S16 Figure. Obviously, this is no proof that the deletion does not confound the overall stability of the protein in vivo. However, our finding that the complementation of the comC knock out mutant with **the truncated versions of ComC** **does restore natural competence to some extent suggests that the deletion mutant of ComC cannot be entirely misfolded.** Moreover, we **now perform complementation with the wild-type mutant of ComC_Var2_** and found that this does neither rescue twitching nor natural competence. Please also see our answers to M3.12 and to M2.1.

### M3.12 - I am not convinced that the experimental results found in Fig. 5 demonstrate specifically the role of the VWA_2 PFAM (nor or the finger) domain in host cell adhesion, twitching motility or competence…

*They may demonstrate some role for the presence of the N-ter of the protein for the functioning of the T4aP, but I believe that by design the experiments cannot address the question on how the “content” of the N-ter may affect function. Some exchanges in N-ter experiments between variants, some point mutations of specific aa, or other experimental designs, may further contribute to address this question. For instance, what happens when performing the exact same experiments* ***with corresponding fragments from the variant v2, or v1-2? Would they or not result in similar phenotypes? This should at least be discussed.***

We thank the reviewer for this comment. We know that our experimental approaches can shed only initial light on the functional differences of the various ComC variants, and on the precise function of the finger-like protrusion as the most prominent difference between the variants. Still, we are convinced that our findings support the claims made in the manuscript. To summarize it:

1. The deletion of comC substantially reduces the capability of *A. baumannii* AYE to adhere to human epithelial cells. **A general defect in piliation is not detected**. This supports our conclusion **that T4aP in general and ComC in particular are mediating the adherence of *A. baumannii* to human cells** as it has been proposed for other pathogens.
2. The deletion of *comC* abolishes twitching and natural competence. This supports our conclusion **that ComC is an essential component for both processes.**
3. A complementation of the knock-out strain with ComC lacking the region showing the similarity to the VWA_2 Pfam model does not rescue host cell adhesion and twitching. It rescues natural competence at least partly, which indicates that the truncated ComC cannot completely mis-fold.
4. A complementation with ComC lacking only the region predicted to fold into the finger-like structure results in the same phenotype as (3). We conclude that the removal of these amino acids is sufficient to functionally disturb ComC. Given that this mutant is predicted to fold into a structure that is comparable to the wild-type ComC except that it lacks the finger (see S16 Figure), we **speculate** that the finger is important for host-cell adherence and twitching.
5. To rule out that an undetected mis-folding of the mutant ComC generates the observed phenotypes, we now complement the comC knock-out strain with Var2 of ComC. This does not rescue the mutant phenotype (see the updated Fig. 5). We conclude that ComC_Var1-1_ and ComC_Var2_ variants are not functionally equivalent. Please see also our answer to M2.1.

To conclude this part, **our experiments show that ComC_Var1-1_ and ComC_Var2_ cannot functionally complement each other**. The most prominent difference lies in the N-terminal part, and here in the region that supposedly generates the finger-like protrusion. Exclusive deletion of this region results in a twitching and adhesion phenotype. This now forms the basis for dedicated follow-up experiments that will help determining the function of this finger with higher precision than in the initial analysis presented here. We make it now clear in the discussion, that the resolution of our experimental approach allows only a tentative functional assignment of the finger.

### MI3.1 - Line 36, abstract section: “Both genes together form an evolutionary cassette” is not totally reflecting the scenario proposed by the authors…

*, because the proposed cassette would contain several extra genes form the T4aP, right?*

This is correct, and we have adjusted the sentence. We now write:

*All three genes are part of an evolutionary cassette (…)*

### MI3.2 - I found the end of introduction too detailed on the results, it is somehow redundant with an abstract.

Motivated by the reviewer’s commente, we have completely re-written the last paragraph of the introduction. **We write now**:

*Therefore, it is still unclear to what extent T4aP differ between members of this genus and what consequences this may have for the interaction of A. baumannii with the human host.*

*Here, we increase the resolution of the comparative analysis of T4aP components to the level of individual protein features, such as the presence of Pfam- or SMART domains, of transmembrane domains and of low complexity regions [14]. For each protein, we integrate the annotated features from N- to C-terminus into a feature architecture and compare these between orthologs of the T4aP components. We then exploit that differences in the feature architectures of two orthologs serve as a proxy of their functional divergence [14]. To thoroughly chart the extent of variation in the precise design of 20 T4aP across the genus Acinetobacter, we integrated their genus-wide phylogenetic profiles across more than 850 bacterial isolates with an assessment of feature architecture similarity. Three candidates organized in the same gene cluster have altered feature architectures in most pathogenic Acinetobacter isolates, which indicates a change in function. A subsequent evolutionary characterization integrated with modelling of their 3D structures and a downstream experimental characterization identifies the pilus tip adhesin ComC as the most prominent candidate driving the functional diversification of T4aP in pathogenic Acinetobacter. In summary, our results provide for the first-time evidence that pathogenic Acinetobacter have modulated the precise function of T4aP by changing the structural layout of the pilus tip adhesin*

### MI3.3 - I do not see the connection with the paragraph on lines 124-137 and the very 1st line of the paragraph…

*"Acinetobacter T4aP components are best characterized in the naturally transformable bacterium A. baylyi ADP1". Should this sentence be placed elsewhere?*

The entire analysis starts with the Acinetobacter T4aP that is best investigated, the T4aP in *A. baylyi*. Here, the individual components have been initially detected, and the model shown in Fig. 1A has been developed. The next step in the analysis is the transfer of this information to *A. baumannii* ATCC 19606. Step. 3 is then the establishment of the phylogenetic profiles for the Ab ATCC 19606 proteins across 884 bacterial taxa. To make this more clear, we have revised the first sentence of this paragraph. **We write now**:

*Acinetobacter T4aP components are best characterized in the naturally transformable bacterium A. baylyi ADP1 (Fig. 1A), and we used the protein set provided by Averhoff et al. 2021 [30] to prime our analysis.*

### MI3.4 - Fig. 1B. it is unclear what should be compared here. Also, I do not get the meaning of the FAS_R score?...

*Why do we need two scores here, and what is “the ortholog”? Please clarify to help the reader analyse the figure.*

Thank you for indicating that our explanation may require too much prior knowledge. Fig. 1B shows the fraction of isolates per species where an ortholog to a T4aP component was detected (dot size). The color code represents the feature architecture similarity (FAS) score of the *Ab* ATCC 19606 protein and of its orthologs per species. The FAS score is **a non-symmetric measure of the similarity between two feature architectures [14]**. In a nutshell, the FAS_F score penalizes features that are present in the Ab ATCC 19606 protein but are not detected in the orthologs. The FAS_R score penalized features that are present in the orthologs but are not detected in the *Ab* ATCC 19606 protein. Please note, we analyzed more than one isolate per species and have summarized the phylogenetic profile on the species level. Hence, we show the median FAS score per species. To address the reviewer’s comment, we have adjusted the caption of Fig. 1. **We write now**:

*A dot indicates the presence of an ortholog in the respective species, and the dot size represents the fraction of the subsumed isolates an ortholog was identified in. The color encodes the median feature architecture similarity (FAS score) between the protein in Ab ATCC 19606 ^T^ and its orthologs within a species. The dot color gradient (FAS_F; blue to orange) captures architecture differences using the feature architecture of the Ab ATCC 19606^T^ protein as reference. The score decreases if features seen in the Ab ATCC 19606^T^ protein are missing in the respective orthologs. The cell color gradient (FAS_B; white to pink) captures architecture differences using the feature architecture of the ortholog as reference. The score decreases if features seen in the ortholog are missing in the Ab ATCC 19606^T^ protein.*

### MI3.5 - Lines 149-150: “the architectures of four proteins, PilX, FimT, FimU, and ComC deviate to an extent between orthologs that it could indicate a change in function.”…

*From Fig. 1B, one could also wonder about the relevance of ComO and ComN.*

We agree with the reviewer that ComO and ComN could be also interesting candidates. Both proteins differ in their feature architecture between isolates, however this difference is caused by low complexity regions and coiled coil domains (see S2 Figure below). The functional relevance of such differences is hard to predict. We therefore concentrated in our analysis on those proteins where the difference in the feature architectures of two orthologs involves at least one Pfam domain. **In our manuscript, we explicitly mention this filter and show the corresponding architectures in S2 Figure, which we provide here for convenience.** We write:

*For most proteins, feature architectures are either conserved across the genus, or differ only in the presence/absence of low complexity regions or coiled coil regions (see S2 Fig).*

*S2 Figure. Feature architecture comparisons of Ab ATCC 19606 T4aP components whose differences between orthologs do not include Pfam or SMART domains. For the three represented components (A-C), the differences in domain architectures between the protein in Ab ATCC 19606 and their orthologs in other isolates are limited to coiled coil regions, low complexity regions (LCR) and regions with a compositional bias (FLPS) (see main text, Fig. 1).* *Reference proteins shown in the first column are compared against their orthologs in Acinetobacter soli CIP 110264 (GCF_000368705.1). TMM – Transmembrane domain; Gln – Glutamine; Ile – Isoleucine.*

### MI3.6 - Line 124: Included in what? Please rephrase as it is unclear.

We have rephrased this sentence to improve clarity. We write now:

*Acinetobacter T4aP components are best characterized in the naturally transformable bacterium A. baylyi ADP1 (Fig. 1A), and we used the sets provided by Averhoff et al., 2021 [30] to prime our analysis. We additionally considered the prepilin peptidase PilD because the corresponding gene is part of the pilBCD operon in A. baylyi, and because it is likely involved in T4aP biogenesis [41, 45].*

### MI3.7 - Line 170-171, it is mentioned that ComC and FimU are close in the genomes of Ab ATCC 19606 T is this also the case in other Acinetobacter genomes?

ComC, FimU and PilV are in close vicinity throughout the genus *Acinetobacter*. We would like to draw the reviewer’s attention to S9 Figure, where we show that the gene cluster harboring ComC, FimU and PilV is conserved throughout the genus. We provide this figure for convenience reasons below.

*S8 Figure. Gene order conservation analysis in the genomic region harboring ComC and FimU. Each line in the plot represents the bacterial isolate that is indicated to the left. The color coding of the taxon labels corresponds to their lineage assignment in Fig. 3 of the main text. Each box represents an ortholog to one of the 13 genes in the ComC/FimU region of Ab ATCC 19606 (see main Fig. 3) and the gene identity is given by the box label. The order of boxes corresponds to the order of genes in the genome of the respective isolate. comC orthologs are marked in white. Green and red boxes identify genes that are upstream and downstream of comC in Ab ATCC 19606, respectively. The number of genes separating any two of the boxed genes or that are placed upstream or downstream up to the scaffold end (indicated by yellow bars) are given in parenthesis. The plot confirms that the gene order in this region is conserved throughout the genus with very few exceptions.*

### MI3.8 - Figure 2: what are the black branches?

We now specify explicitly that black branches represent isolates from all other species that are not *A. baumannii.* **We write now**:

***Figure 2. Maximum likelihood phylogeny of ComC.*** *Red branches indicate A. baumannii isolates and black branches isolates from all other species*

### MI3.9 - Line 185-186: “This indicates that recombination has affected the evolution of this locus”…

*I am not sure this can be argued at this point of the text. “might have affected” could be more appropriate?*

We agree with the reviewer that we should stay vague here. **We write now:**

*This suggests that recombination may have affected the evolution of this locus.*

### MI3.10 - Line 386: "RefSeq database release 204" Could the authors mention on what date it was last accessed?

March 07, 2021. We have now added this information to the Materials and Methods section.

### Typos

#### Typo1 - Line 74 : remove «how »

Done

#### Typo2 - Line324: PDB means “Protein Data Bank”. Authors may want to add in text it is a database of structures of proteins?

Done

#### Typo3 - Line 413: where => were

Done

## References

1.	Higgins PG, Dammhayn C, Hackel M, Seifert H. Global spread of carbapenem-resistant Acinetobacter baumannii. The Journal of antimicrobial chemotherapy. 2010;65(2):233-8. doi: 10.1093/jac/dkp428. PubMed PMID: 19996144.

2.	Murray CJL, Ikuta KS, Sharara F, Swetschinski L, Aguilar GR, Gray A, et al. Global burden of bacterial antimicrobial resistance in 2019: a systematic analysis. Lancet. 2022;399(10325):629-55. Epub 2022/01/24. doi: 10.1016/S0140-6736(21)02724-0. PubMed PMID: 35065702; PubMed Central PMCID: PMCPMC8841637.

3.	Tietgen M, Kramer JS, Brunst S, Djahanschiri B, Wohra S, Higgins PG, et al. Identification of the novel class D beta-lactamase OXA-679 involved in carbapenem resistance in Acinetobacter calcoaceticus. The Journal of antimicrobial chemotherapy. 2019;74(6):1494-502. Epub 2019/03/08. doi: 10.1093/jac/dkz080. PubMed PMID: 30844059.

4.	Djahanschiri B, Di Venanzio G, Distel JS, Breisch J, Dieckmann MA, Goesmann A, et al. Evolutionarily stable gene clusters shed light on the common grounds of pathogenicity in the Acinetobacter calcoaceticus-baumannii complex. PLoS Genet. 2022;18(6):e1010020. Epub 2022/06/03. doi: 10.1371/journal.pgen.1010020. PubMed PMID: 35653398; PubMed Central PMCID: PMCPMC9162365.

5.	Williams KP, Gillespie JJ, Sobral BW, Nordberg EK, Snyder EE, Shallom JM, et al. Phylogeny of gammaproteobacteria. J Bacteriol. 2010;192(9):2305-14. Epub 2010/03/09. doi: 10.1128/JB.01480-09. PubMed PMID: 20207755; PubMed Central PMCID: PMCPMC2863478.

6.	Eijkelkamp BA, Stroeher UH, Hassan KA, Paulsen IT, Brown MH. Comparative analysis of surface-exposed virulence factors of Acinetobacter baumannii. BMC Genomics. 2014;15(1):1020. Epub 2014/11/26. doi: 10.1186/1471-2164-15-1020. PubMed PMID: 25422040; PubMed Central PMCID: PMCPMC4256060.

7.	Treuner-Lange A, Chang YW, Glatter T, Herfurth M, Lindow S, Chreifi G, et al. PilY1 and minor pilins form a complex priming the type IVa pilus in Myxococcus xanthus. Nature communications. 2020;11(1):5054. Epub 2020/10/09. doi: 10.1038/s41467-020-18803-z. PubMed PMID: 33028835; PubMed Central PMCID: PMCPMC7541494.

8.	Piepenbrink KH, Lillehoj E, Harding CM, Labonte JW, Zuo X, Rapp CA, et al. Structural Diversity in the Type IV Pili of Multidrug-resistant Acinetobacter. J Biol Chem. 2016;291(44):22924-35. Epub 2016/10/30. doi: 10.1074/jbc.M116.751099. PubMed PMID: 27634041; PubMed Central PMCID: PMCPMC5087714.

9.	Dosch J, Bergmann H, Tran V, Ebersberger I. FAS: Assessing the similarity between proteins using multi-layered feature architectures. bioRxiv. 2022:2022.09.01.506207. doi: 10.1101/2022.09.01.506207.

10.	Humphrey W, Dalke A, Schulten K. VMD: visual molecular dynamics. J Mol Graph. 1996;14(1):33-8, 27-8. Epub 1996/02/01. doi: 10.1016/0263-7855(96)00018-5. PubMed PMID: 8744570.

11.	Roberts E, Eargle J, Wright D, Luthey-Schulten Z. MultiSeq: unifying sequence and structure data for evolutionary analysis. BMC Bioinformatics. 2006;7:382. Epub 2006/08/18. doi: 10.1186/1471-2105-7-382. PubMed PMID: 16914055; PubMed Central PMCID: PMCPMC1586216.

12.	Russell RB, Barton GJ. Multiple protein sequence alignment from tertiary structure comparison: assignment of global and residue confidence levels. Proteins. 1992;14(2):309-23. Epub 1992/10/01. doi: 10.1002/prot.340140216. PubMed PMID: 1409577.

13.	Tunyasuvunakool K, Adler J, Wu Z, Green T, Zielinski M, Zidek A, et al. Highly accurate protein structure prediction for the human proteome. Nature. 2021;596(7873):590-6. Epub 2021/07/23. doi: 10.1038/s41586-021-03828-1. PubMed PMID: 34293799; PubMed Central PMCID: PMCPMC8387240.

14.	Dosch J, Bergmann H, Tran V, Ebersberger I. FAS: Assessing the similarity between proteins using multi-layered feature architectures. Bioinformatics. 2023. Epub 2023/04/21. doi: 10.1093/bioinformatics/btad226. PubMed PMID: 37084276.

15.	Ebersberger I, Strauss S, von Haeseler A. HaMStR: Profile hidden markov model based search for orthologs in ESTs. BMC Evolutionary Biology. 2009;9(1):157. doi: 10.1186/1471-2148-9-157.

16.	Ostlund G, Schmitt T, Forslund K, Kostler T, Messina DN, Roopra S, et al. InParanoid 7: new algorithms and tools for eukaryotic orthology analysis. Nucleic Acids Res. 2010;38(suppl\_1):D196-203.

17.	Altenhoff AM, Levy J, Zarowiecki M, Tomiczek B, Warwick Vesztrocy A, Dalquen DA, et al. OMA standalone: orthology inference among public and custom genomes and transcriptomes. Genome Res. 2019;29(7):1152-63. Epub 2019/06/27. doi: 10.1101/gr.243212.118. PubMed PMID: 31235654; PubMed Central PMCID: PMCPMC6633268.

18.	Nevers Y, Jones TEM, Jyothi D, Yates B, Ferret M, Portell-Silva L, et al. The Quest for Orthologs orthology benchmark service in 2022. Nucleic Acids Res. 2022;50(W1):W623-W32. Epub 2022/05/14. doi: 10.1093/nar/gkac330. PubMed PMID: 35552456; PubMed Central PMCID: PMCPMC9252809.

19.	Altenhoff AM, Garrayo-Ventas J, Cosentino S, Emms D, Glover NM, Hernandez-Plaza A, et al. The Quest for Orthologs benchmark service and consensus calls in 2020. Nucleic Acids Res. 2020;48(W1):W538-W45. Epub 2020/05/07. doi: 10.1093/nar/gkaa308. PubMed PMID: 32374845; PubMed Central PMCID: PMCPMC7319555.

20.	Birikmen M, Bohnsack KE, Tran V, Somayaji S, Bohnsack MT, Ebersberger I. Tracing Eukaryotic Ribosome Biogenesis Factors Into the Archaeal Domain Sheds Light on the Evolution of Functional Complexity. Frontiers in microbiology. 2021;12. doi: 10.3389/fmicb.2021.739000.

21.	Finn RD, Clements J, Eddy SR. HMMER web server: interactive sequence similarity searching. Nucleic Acids Res. 2011;39(Web Server issue):W29-37. doi: 10.1093/nar/gkr367. PubMed PMID: 21593126; PubMed Central PMCID: PMCPMC3125773.

22.	Mistry J, Chuguransky S, Williams L, Qureshi M, Salazar GA, Sonnhammer ELL, et al. Pfam: The protein families database in 2021. Nucleic Acids Res. 2021;49(D1):D412-D9. Epub 2020/10/31. doi: 10.1093/nar/gkaa913. PubMed PMID: 33125078; PubMed Central PMCID: PMCPMC7779014.

23.	Letunic I, Bork P. 20 years of the SMART protein domain annotation resource. Nucleic Acids Res. 2018;46(D1):D493-D6. Epub 2017/10/19. doi: 10.1093/nar/gkx922. PubMed PMID: 29040681; PubMed Central PMCID: PMCPMC5753352.

24.	Emsley J, Cruz M, Handin R, Liddington R. Crystal structure of the von Willebrand Factor A1 domain and implications for the binding of platelet glycoprotein Ib. J Biol Chem. 1998;273(17):10396-401. Epub 1998/05/30. doi: 10.1074/jbc.273.17.10396. PubMed PMID: 9553097.

25.	Gibrat JF, Madej T, Bryant SH. Surprising similarities in structure comparison. Curr Opin Struct Biol. 1996;6(3):377-85. Epub 1996/06/01. doi: 10.1016/s0959-440x(96)80058-3. PubMed PMID: 8804824.

26.	Siryaporn A, Kuchma SL, O'Toole GA, Gitai Z. Surface attachment induces Pseudomonas aeruginosa virulence. Proc Natl Acad Sci U S A. 2014;111(47):16860-5. doi: 10.1073/pnas.1415712111. PubMed PMID: 25385640; PubMed Central PMCID: PMCPMC4250119.

27.	Lee JO, Rieu P, Arnaout MA, Liddington R. Crystal structure of the A domain from the alpha subunit of integrin CR3 (CD11b/CD18). Cell. 1995;80(4):631-8. Epub 1995/02/24. doi: 10.1016/0092-8674(95)90517-0. PubMed PMID: 7867070.

28.	Shimodaira H, Hasegawa M. Multiple Comparisons of Log-Likelihoods with Applications to Phylogenetic Inference. Mol Biol Evol. 1999;16(8):1114-6.

29.	Mostowy R, Croucher NJ, Andam CP, Corander J, Hanage WP, Marttinen P. Efficient Inference of Recent and Ancestral Recombination within Bacterial Populations. Mol Biol Evol. 2017;34(5):1167-82. Epub 2017/02/16. doi: 10.1093/molbev/msx066. PubMed PMID: 28199698; PubMed Central PMCID: PMCPMC5400400.

30.	Averhoff B, Kirchner L, Pfefferle K, Yaman D. Natural transformation in Gram-negative bacteria thriving in extreme environments: from genes and genomes to proteins, structures and regulation. Extremophiles. 2021;25(5-6):425-36. Epub 2021/09/21. doi: 10.1007/s00792-021-01242-z. PubMed PMID: 34542714; PubMed Central PMCID: PMCPMC8578077.
